# *DICER1* tumor predisposition syndrome: an evolving story initiated with the pleuropulmonary blastoma

**DOI:** 10.1038/s41379-021-00905-8

**Published:** 2021-10-01

**Authors:** Iván A. González, Douglas R. Stewart, Kris Ann P. Schultz, Amanda P. Field, D. Ashley Hill, Louis P. Dehner

**Affiliations:** 1 Department of Pathology and Laboratory Medicine, Children’s Hospital of Philadelphia, Philadelphia, PA USA; 2 Clinical Genetics Branch, Division of Cancer Epidemiology and Genetics, National Cancer Institute, Rockville, MD USA; 3 International Pleuropulmonary Blastoma/DICER1 Registry, Children’s Minnesota, Minneapolis, MN USA; 4 Cancer and Blood Disorders, Children’s Minnesota, Minneapolis, MN USA; 5 ResourcePath LLC, Sterling, VA USA; 6 Division of Pathology, Children’s National Medical Center, George Washington University School of Medicine and Health Sciences, Washington, DC USA; 7 The Lauren V. Ackerman Laboratory of Surgical Pathology, Barnes-Jewish and St. Louis Children’s Hospitals, Washington University Medical Center, St. Louis, MO USA

**Keywords:** Cancer genetics, Paediatric cancer

## Abstract

*DICER1* syndrome (OMIM 606241, 601200) is a rare autosomal dominant familial tumor predisposition disorder with a heterozygous *DICER1* germline mutation. The most common tumor seen clinically is the pleuropulmonary blastoma (PPB), a lung neoplasm of early childhood which is classified on its morphologic features into four types (IR, I, II and III) with tumor progression over time within the first 4–5 years of life from the prognostically favorable cystic type I to the unfavorable solid type III. Following the initial report of PPB, its association with other cystic neoplasms was demonstrated in family studies. The detection of the germline mutation in *DICER1* provided the opportunity to identify and continue to recognize a number seemingly unrelated extrapulmonary neoplasms: Sertoli-Leydig cell tumor, gynandroblastoma, embryonal rhabdomyosarcomas of the cervix and other sites, multinodular goiter, differentiated and poorly differentiated thyroid carcinoma, cervical-thyroid teratoma, cystic nephroma-anaplastic sarcoma of kidney, nasal chondromesenchymal hamartoma, intestinal juvenile-like hamartomatous polyp, ciliary body medulloepithelioma, pituitary blastoma, pineoblastoma, primary central nervous system sarcoma, embryonal tumor with multilayered rosettes-like cerebellar tumor, PPB-like peritoneal sarcoma, *DICER1*-associated presacral malignant teratoid neoplasm and other non-neoplastic associations. Each of these neoplasms is characterized by a second somatic mutation in *DICER1*. In this review, we have summarized the salient clinicopathologic aspects of these tumors whose histopathologic features have several overlapping morphologic attributes particularly the primitive mesenchyme often with rhabdomyoblastic and chondroid differentiation and an uncommitted spindle cell pattern. Several of these tumors have an initial cystic stage from which there is progression to a high grade, complex patterned neoplasm. These pathologic findings in the appropriate clinical setting should serve to alert the pathologist to the possibility of a *DICER1*-associated neoplasm and initiate appropriate testing on the neoplasm and to alert the clinician about the concern for a *DICER1* mutation.

## Introduction

*DICER1* gene is located on chromosome 14q32.13 and plays a crucial role in the control of protein translation; its product, dicer protein, is a ribonuclease (RNase) III endoribonuclease which is essential for the production of microRNAs (miRNA) which are formed by the cleavage of pre-miRNA or double-stranded RNA^1–4^. RNase III contains two domains, IIIa and IIIb which cleave 3p miRNA and 5p miRNA from the 3′ and 5′ pre-miRNA, respectively. These cleavages require magnesium ions at the interface between the IIIa and IIIb domains and the miRNA; this magnesium dependent catalytic processing occurs at specific residues, E1320, E1564, E1813 and D1709^2–4^. miRNA has a pivotal role in regulating the expression of over 30% of protein-coding genes by its interaction with mRNA^5^. Given the impact of *DICER1* in post-translational events, it is not entirely surprising that functional *DICER1* is essential for vertebrate development as evidenced by developmental arrest and death of the embryo when both alleles are lost^6,7^. Conceptually, *DICER1* can be regarded as either a tumor suppressor gene due to loss-of-function mutations or an oncogene due to gain-of-function mutations; it is thought to function as a haploinsufficient tumor suppressor gene with the loss of one allele leading to tumor progression but loss of both alleles having an inhibitory effect for tumor development implying that one intact allele is needed for cell survival^8^.

A study led by one of the authors (DAH) identified germline loss-of-function *DICER1* mutations affecting the RNase IIIb domain in affected families with pleuropulmonary blastoma (PPB)^9^, a rare dysembryonic lung malignancy of childhood which was not the only manifestation of this familial tumor predisposition syndrome; germline and somatic *DICER1* mutations were subsequently identified in several other familial associated tumors in several extrapulmonary sites (Table 1). Individuals with germline *DICER1* mutations also had non-neoplastic conditions including macrocephaly, renal structural anomalies, retinal abnormalities, dental perturbations, and the GLOW syndrome (global developmental delay, lung cysts, overgrowth and Wilms tumor). These associations encircle the *DICER1* tumor predisposition syndrome (Online Mendelian Inheritance in Man numbers 606241, 601200 and 138800), with the estimation that 90% of those affected by this syndrome inherited a germline mutation from one of their parents, with a pattern of autosomal dominant inheritance^10^.

**Table 1:** *DICER1*-associated neoplasms.

Pleuropulmonary blastoma (PPB) and PPB-like neoplasms
Pleuropulmonary blastoma, type I, IR, II, III
PPB-like Sertoli-Leydig cell tumor of lung
Pediatric cystic neoplasms and *DICER1*-sarcoma (anaplastic sarcoma of kidney)
Nasal chondromesenchymal hamartoma
Central nervous system sarcoma with rhabdomyosarcoma/PPB III-like features
Sertoli-Leydig cell tumor with and without heterologous features and type I PPB-like features
Peritoneal, ovarian and fallopian tube sarcoma with PPB-like features
*DICER1*-associated cystic hepatic neoplasm with type I PPB-like features
Cervical embryonal rhabdomyosarcoma
Teratoid and primitive neuroepithelial neoplasms
Cervical-thyroid teratoma
Malignant teratoid neoplasm of sacrococcygeal region
Ciliary body medulloepithelioma
Pituitary blastoma
Pineoblastoma
Embryonal tumor with multilayered rosettes
Thyroid
Multinodular hyperplasia (goiter)
Papillary thyroid carcinoma, invasive follicular variant
Follicular carcinoma, pediatric type
Poorly differentiated thyroid carcinoma, pediatric type
Intestine
Hamartomatous polyp with juvenile polyp-like features

Most of the tumors in this syndrome occur in affected individuals with one inherited *DICER1* mutation, mainly a loss-of-function mutation, and an acquired somatic missense *DICER1* mutation within five hotspot codons in the RNase IIIb domain (E1705, D1709, G1809, D1810 and E1813)^11–14^. These variants result in a bias toward the production of 3p strands of miRNA with loss of 5p strands of miRNA^15^. Although this is the most common mutational pattern in *DICER1*-associated tumors, mosaicism for missense variants in these same hotspot codons have also been identified and are associated with a more severe phenotype; mosaicism explains those cases of PPB and the other associated tumors in a child without a *DICER1* germline mutation^10^. The estimated prevalence of pathogenic *DICER1* variants in the general population is ~1:10,600, and approximately 30,000 Americans harbor pathogenic *DICER1* mutations^12,14^; however, this prevalence is seemingly higher and is estimated at ~1:4600 in the adult cancer population^14^.

Following the initial clinicopathologic report of the PPB as a unique high grade, solid neoplasm of the lung presenting early in the first decade of life^16^, it was not until the International PPB Registry (IPPBR) was established with the availability to study additional cases that an apparent familial predisposition was recognized as well as the occurrence of other extrapulmonary tumors in these kindreds^17^. Later came the identification of *DICER1* variants in these affected kindreds^9^. After more than three decades of this seminal observation^16^, numerous studies have confirmed the relationship between *DICER1* variants in carriers and the development of a range of neoplasms and non-neoplastic conditions; these associations have served to clarify the molecular genetic nature of previously known pathologic entities such as cystic nephroma and Sertoli-Leydig cell tumor, but also to identify and characterize entities which were included among other apparently unrelated neoplastic processes or not recognized as familial associated pathology^18^. Despite the disparate primary sites of the *DICER1*-associated neoplasms, many of these tumors have overlapping pathologic features to possibly reflect their origin in sites of branching morphogenesis (lung, kidney, liver), a key developmental role of *DICER1*^19,20^. It had been noted earlier that the conditional *DICER1* knockout in the developing mouse lung resulted in the formation of cysts with the failure in branching morphogenesis; this observation served as the foundation for the hypothesis that a *DICER1* mutation may explain the morphogenesis of the initial cystic stage of PPB^9,21^.

## Pleuropulmonary blastoma (PPB)

**Fig. 1:**
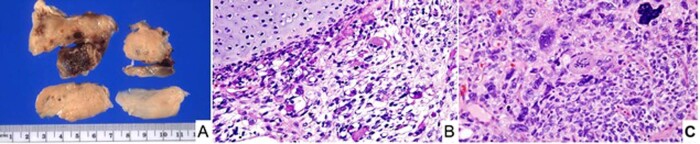
Pleuropulmonary blastoma, type III. **A** Pleuropulmonary blastoma, type III occurring in a 3-year-old male as a large solid thoracic mass. Piecemeal resection revealed a tan-white, myxoid and hemorrhagic neoplasm (one of the original 11 PPB cases). **B** Focus of embryonal type rhabdomyosarcoma and nodule of malignant cartilage. **C** Another focus of primitive sarcoma with individual anaplastic cells including bizarre mitotic figure.

Considerable progress has been made since the first cases of PPB were described almost 45 years ago when it was proposed that this neoplasm was the nosologic equivalent to the other dysembryonic neoplasms of childhood such as neuroblastoma and Wilms tumor unlike the extant classic biphasic pulmonary blastoma of Spencer which was a neoplasm predominantly of adults^22^. The initial 11 cases of PPB were all large solid masses arising in the lung and/or pleura and were composed in part of embryonal-type rhabdomyosarcoma, nodules of chondrosarcoma, primitive spindle and small round cells with blastemal features and anaplasia; these tumors in retrospect were all examples of the solid or type III PPB (Fig.1A–C)^16^. It was only later with additional cases referred to the IPPBR that it appeared that the solid tumor was the ultimate stage in the process of tumor progression from a multicystic lung lesion, previously regarded as a congenital pulmonary airway malformation (CPAM) type IV, to an intermediate mixed cystic and solid stage whose latter pathologic features were those of the originally reported high grade solid PPB^23^.

Two points in our evolving understanding of PPB set the stage for the subsequent recognition of a tumor predisposition syndrome: one was the report from the IPPBR of 45 patients with PPB that revealed that 12 (27%) children had first and second degree relatives with other conditions including pulmonary “cysts”, cystic nephroma, thyroid nodules and neoplasms and rhabdomyosarcoma (RMS), among other tumors presenting in the first two decades of life^17^ and the second observation was the existence of a morphologic spectrum of PPB that correlated with age at diagnosis and clinical outcome^23^. This morphologic transition ranged from a cystic lung lesion, typically recognized in the first year of life if not *in utero*, which was designated type I (cystic) PPB, to a penultimate cystic and solid type II and the ultimate solid type III PPB, the pathologic type identified in the original 11 cases^16^. This temporal evolution from a cystic to a solid tumor also allowed for the study of those factors involved in the progression from a type I to type III PPB^24^. The latter observation carried considerable importance given the excellent outcome of type I PPB with a 5-year overall survival (OS) of over 90% to a 5-year OS of 71% and 53% in the case of type II and type III PPB, respectively^25^.

**Fig. 2:**
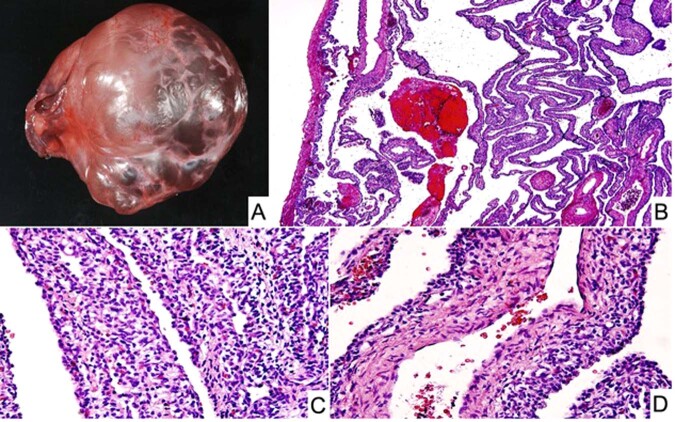
Pleuropulmonary blastoma, type I presenting as a cystic lesion in the lung of a 1-year-old female. **A** Septal markings and cysts are appreciated in this unbisected specimen whose multicystic structure is lost with bisection as the delicate septa collapse. **B** The peripheral multicystic lesion is composed of uniform septal structures abutting the pleural surface, a characteristic feature. **C** Uniformly expanded septa by primitive-appearing small, rounded and spindled-shaped cells, many showing desmin and myogenin positivity (not shown). **D** Cambium layer-like concentration of primitive small cells with adjacent fibrous stroma.

**Fig. 3:**
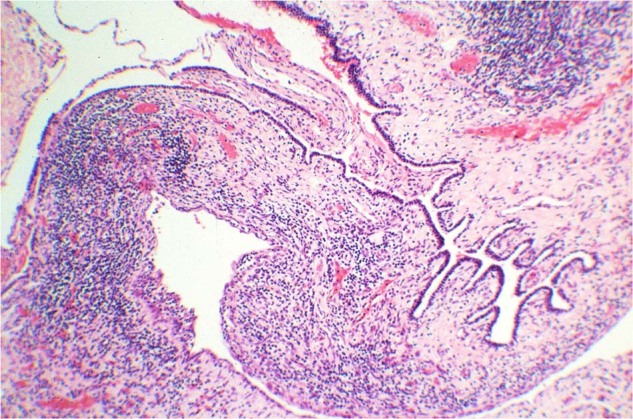
Pleuropulmonary blastoma, type I showing septal widening. Pleuropulmonary blastoma, type I showing a focus of septal widening by primitive small cells as a feature of presumed progression before the formation of a grossly visible mass, as a requisite for the diagnosis of type II PPB.

**Fig. 4:**
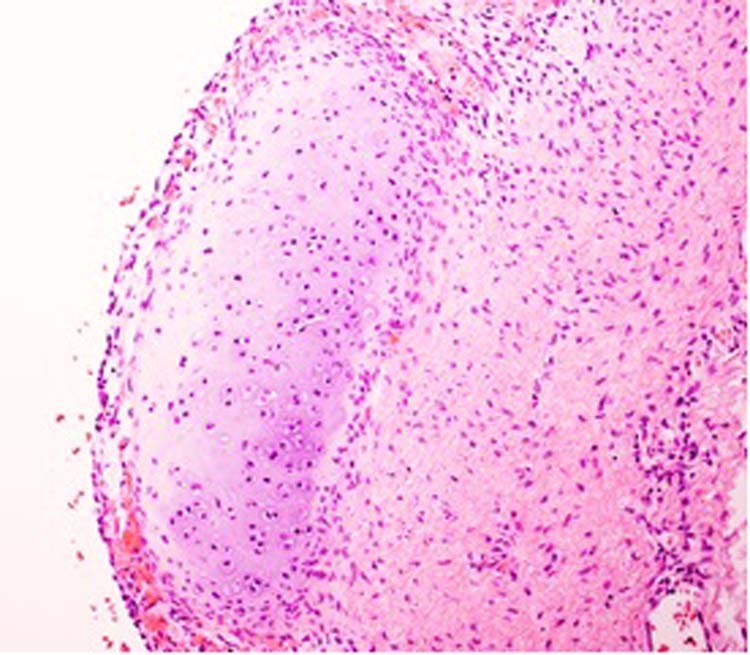
Pleuropulmonary blastoma, type I showing a nodule of cartilage within a septum. This finding is seen in 15% of type I PPBs, as one or several foci.

Given the prognostic implication of the PPB type, an accurate diagnosis and proper classification, particularly in the case of type I PPB with its potential erroneous interpretation as a CPAM, are of utmost importance for appropriate clinical management. In more detail, type I PPB is a multicystic well-demarcated lesion arising from the peripheral or distal sac-like structures lined by a flat to low cuboidal epithelium and a variably collagenized stroma with a fine capillary network, and a discontinuous or continuous subepithelial layer of small primitive round cells with or without rhabdomyoblastic differentiation with a so-called cambium-like layer appearance (Fig. 2A–D). In some cases scattered nodules of cartilage with a fetal rather than sarcomatous appearance are also present within the septa (Fig. 3)^22–24^. The small primitive neoplastic cells may be confined to the subepithelial zone or expand and replace the background fibrovascular stroma without the formation of a grossly visible mass, a requisite feature of type II PPB (Fig. 4). The cellularity of the septa can vary from hypo- to hypercellular so that complete sampling of the cystic lesion is necessary since the small primitive cells and/or rhabdomyoblasts are focal with a limited distribution within the septa or are more diffusely cellular and distributed to facilitate identification so that it is important to examine the entire specimen microscopically. Regardless of the degree and extent of septal cellularity and expansion, the absence of a grossly detectable mass differentiates a type I from a type II PPB; microscopic features alone do not differentiate a type I from a type II PPB in the absence of a mass. The neoplastic cells may demonstrate apparent rhabdomyoblastic differentiation or may require immunohistochemistry (IHC) for desmin and myogenin. In the absence of a rhabdomyoblastic phenotype, the small cells are diffusely positive for CD56 whose presence still qualify as a type I PPB. A subset of otherwise architecturally similar multicystic lesion lacking the primitive round cells after a thorough examination represents an example of type IR PPB to imply that the cystic PPB has either failed to undergo tumor progression or possibly undergone regression^24^.

**Fig. 5:**
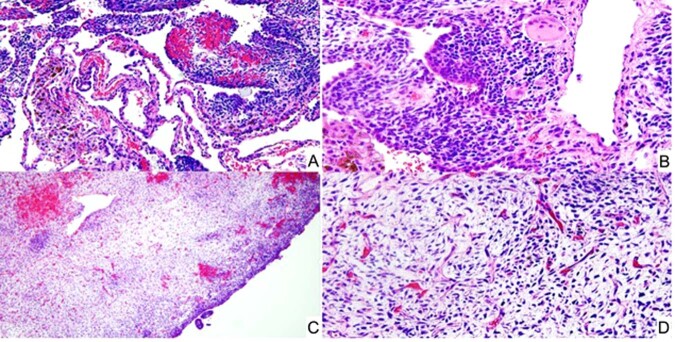
Pleuropulmonary blastoma, type II. **A** Focus of PPB type I in a mass lesion in this 2-year-old male whose tumor was predominantly solid. **B**, **C** A cambium layer-like growth pattern in this transitional area from type I to the solid pattern. **D** Solid focus showing a mixture of small primitive round and spindle cells.

Type II and III PPBs in contrast are mass lesions, unlike type I PPB, and have a solid high-grade pattern which is characterized by a collage of primitive sarcomatous patterns including RMS with embryonal features, nodules of fetal and/or sarcomatous cartilage, islands and nests of compact blastema, primitive spindle cells and scattered or groups of anaplastic cells (Fig. 5)^19^. The solid areas and their composition of patterns vary from one tumor to another in terms of one or more of these several patterns (Fig. 1B, C). The pathologic diagnosis does not require the presence of each one of these several patterns within any one particular tumor; some tumors are dominated by one or another pattern so as to potentially create some uncertainty as to whether a particular neoplasm is a PPB, especially in a biopsy with its restricted sample as to the various patterns. In some cases, molecular studies may be necessary to determine the *DICER1* status; however, the clinical presentation should be correlated with the biopsy. The opportunity to appreciate the various patterns is often deferred since the surgical resection of the lung is currently preceded by neoadjuvant chemotherapy, unlike the earlier cases with primary surgical resection. Type II PPB is differentiated from type III by the presence of residual microscopic cystic foci of type I PPB in addition to a mass; however, a biopsy may only show the solid pattern and does not permit differentiation of a type II from type III PPB since the type I pattern is only appreciated in the post-treatment resection. The identification of residual cystic foci is important since the OS is significantly enhanced in the case of type II PPB compared to type III^25^.

Given the rarity of PPB and the range of morphologic features, it is not altogether surprising that approximately 20% of the cases sent to the IPPBR are not PPBs, but true congenital lung cysts, cystic pleuropulmonary synovial sarcoma and other solid neoplasms such as Ewing sarcoma and even sarcomatoid carcinoma^25^. In the presence of an exclusive spindle cell sarcomatous pattern in a lung neoplasm from an older child or adolescent with the question of a PPB, synovial sarcoma should be considered^26^. This latter experience highlights the desirability of a central review of an extraordinarily rare tumor such as the PPB.

Harris et al.^21^, provided the foundation that *DICER1* may explain the cystic stage since its inactivation in the mouse model resulted in lung cysts resembling type I PPB. A genetic study of 11 affected families with PPBs and other tumors revealed a heterozygous *DICER1* germline mutation with loss-of-function mutation affecting the RNase IIIb^9^. It is currently estimated that at least 70% of patients with a PPB have a germline *DICER1* variant^25^. Mosaicism for RNase IIIb domain missense mutations is associated with a more aggressive phenotype^10,27^. An explanation for this apparent phenotypic difference from the germline cases remains unclear to date.

**Fig. 6:**
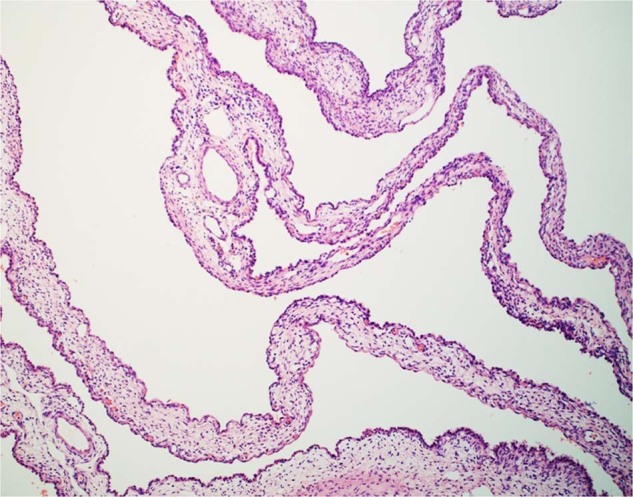
Pleuropulmonary blastoma, type IR. Pleuropulmonary blastoma, type IR showing the characteristic multicystic architecture of type I, but in the absence of a primitive small cell or rhabdomyoblastic population only ascertained after a complete microscopic examination of the resected cyst.

It was appreciated that biallelic *DICER1* mutations were probably insufficient for the progression of type I PPB to types II or III and that an additional mutation(s) was probably necessary and these include *TP53* and *NRAS* mutations^10,28–31^. This observation may explain in part that a subset of type I PPBs lacks the intrinsic potential to progress beyond the purely cystic stage without rhabdomyoblasts and/or small primitive cells as in type IR PPB (Fig. 6)^21^. More recently, it has been demonstrated that TP53 expression by IHC correlates with the prognosis in PPB independent of the three PPB types; this observation validated the hypothesis that other mutations are necessary for tumor progression from type I to type III PPB^32^. Furthermore, it was noted in the latter study that the presence of TP53 expression in the epithelial cells lining the cystic structures in most type II PPBs but in less than 50% of type I PPB and in less than 20% of type IR raised the question of the role that the epithelium plays in tumor progression independent of mutations in the mesenchymal-stromal cells.

Murray et al, on the basis of the increased 3p miRNA hypothesized that the serum levels of miRNA could be used as a screening test for PPB^33^. Increased serum miRNA levels at the time of PPB diagnosis was detected in a patient with a germline *DICER1* mutation which decreased after treatment with chemotherapy. It is still unclear at present what are the implications or the utility of using serum levels of miRNA as either a screening tool, biomarker or as a possible option for follow-up. A prospective population-based study is needed to further answer these questions^34^.

On the basis of our current understanding of *DICER1* and PPB, the IPPBR recommends that a chest x-ray should be obtained for all at risk children at the time of birth to screen for lung cyst(s). Children with a proven germline mutation should have a chest computed tomography (CT) by 9-months of age for a type I PPB so that resection can be performed before possible progression to PPB type II/III^31^. If the chest CT is normal, a follow-up low dose scan is recommended at 2.5-years of age which is prior to the peak incidence of type II and III PPB^35^. It is clear that not all type I PPBs progress to the clinically more aggressive type II/III PPB since type IR PPB is regarded as the failure of tumor progression beyond type I PPB. However, it is necessary to resect the multicystic lesion to determine whether it is type I or type IR PPB (Fig. 6).

## The female reproductive system

Germline pathogenic mutations in *DICER1* are associated with different tumor types affecting the female reproductive system; the two most common are Sertoli-Leydig cell tumors (SLCT) and cervical embryonal rhabdomyosarcoma (cERMS). Merideth et al.^36^, reported on their findings in women *DICER1* carriers from the non-neoplastic gynecologic perspective. Among *DICER1* carriers the mean age of menarche was 12.7 years (range: 10–16 years) with no reports of precocious puberty. Among those women with a history of prior chemotherapy and/or radiation due to a prior PPB, the age of menarche was similar to the general population^36^. All 64 *DICER1* carriers included in the latter study had a normal female phenotype and normal Tanner staging for age. Of the pregnancies identified in this population, 21% resulted in spontaneous miscarriage, 3.6% in first trimester termination, one (0.9%) ectopic pregnancy, and 75% completed pregnancies with only 5% resulting in preterm delivery. Notably, 10 of the 32 (31%) patients that conceived experienced pregnancy-related goiter which resulted in a thyroidectomy within one-year of pregnancy^36^. This study highlights the importance of the awareness of the spectrum of gynecologic and obstetric findings in *DICER1* carriers, especially the pregnancy-related thyroid enlargement as an established manifestation of *DICER1* tumor predisposition syndrome^37^.

### Ovarian sex cord-stromal tumors (OSCST)

OSCSTs are a heterogenous groups of tumors representing approximately 5% of all primary ovarian neoplasms which can present in adolescence and young adulthood, if not earlier as in the case of the juvenile granulosa cell tumor. These tumors are classified according to pure stromal types (Leydig cell tumor, steroid cell tumor), pure sex cord types (adult and juvenile granulosa cell tumors, Sertoli cell tumor) and mixed sex cord-stromal types (Sertoli-Leydig cell tumor, gynandroblastoma)^38^. In addition to an adnexal mass, they may have functional signs due to hormonal production such as hirsutism and virilization, menstrual changes or precocious pseudo-puberty^39,40^. Importantly, OSCSTs are also associated with other underlying predisposition syndromes such as Peutz-Jeghers and Ollier-Maffucci syndromes^41–43^.

The International Ovarian and Testicular Stromal Tumor Registry (IOTSTR) reported that SLCT was one of the more common *DICER1*-associated neoplasms and the most common ovarian tumor overall in the *DICER1* syndrome^44–53^. Germ cell tumors have also been reported in family members of those with a PPB, including a dysgerminoma and three seminomas^49^. Slade et al identified only one case of germ cell tumor, a seminoma, with a biallelic *DICER1* mutation among 172 germ cell tumors^5^. Other ovarian tumors associated with *DICER1* mutations include juvenile granulosa cell tumor, yolk sac tumor, teratomas and mixed germ cell tumors; these tumors compared to SLCT may represent coincidences since it is uncertain whether *DICER1* carriers are at an increased risk for germ cell tumors^46,50,51^.

#### Sertoli-Leydig cell tumor (SLCT)

**Fig. 7:**
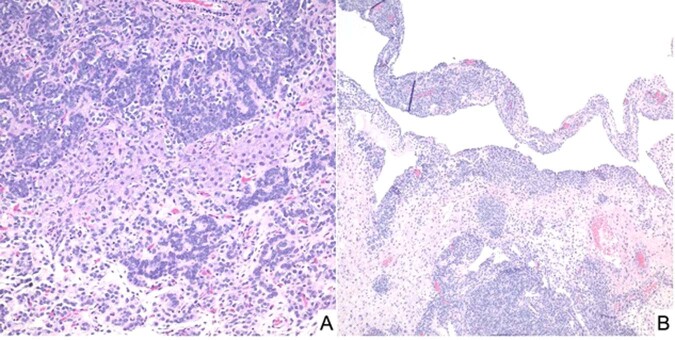
Sertoli-Leydig cell tumor, moderately differentiated in an adolescent presenting with an abdominal mass. **A** Solid foci of this solid and cystic mass showing groups of Sertoli cells and surrounding Leydig cells. **B** Cystic focus with a delicate septum with a cambium layer-like localization of Sertoli cells with its resemblance to PPB type I.

**Fig. 8:**
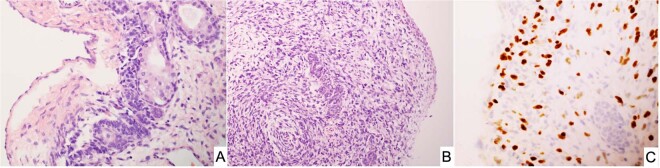
Sertoli-Leydig cell tumor, moderately differentiated in a 15-year-old female. **A** A cystic focus showing a group of Sertoli cells beneath a low cuboidal epithelial surface. **B** Nests of Sertoli cells surrounded by malignant-appearing, primitive-appearing spindle and round cells in another cystic area of the tumor. **C** MyoD immunostaining showing intense nuclear positivity to corroborate heterologous rhabdomyosarcoma.

SLCT represents less than 1% of all primary ovarian tumors and is seen in a broad age range from infancy into the later adult years, but approximately 75% of cases present in women less than 30-years of age^54^. It was appreciated over 50 years ago that there was of an apparent association between the SLCT, multinodular goiter and cERMS^54–58^. In a cohort of 64 cases of intermediate and poorly differentiated SLCT, three patients had a history of a thyroid nodule or goiter^55^. Young and Scully also noted that two patients had a cERMS and four others had a thyroid “abnormality” in their study of ovarian SLCTs, to suggest that this association was more common in those women with an intermediate and poorly-differentiated SLCTs^54^. Once the linkage of *DICER1* and familial PPB cases was reported in 2009^9^, subsequent studies documented the linkage of *DICER1* with SLCT as well as with FOXL2^45^. Pathologically, most SLCTs in the *DICER1* setting have been moderately (intermediate) or poorly differentiated tumors (Fig. 7A, B); some of these neoplasms have had heterologous elements including nodules of cartilage and rhabdomyosarcoma; the latter combination of patterns has been observed in SLCT in addition to PPB as already noted and in other *DICER1*-associated tumors (Fig. 8A–C)^23,59^. The SLCT may have a localized multicystic pattern with more than a passing resemblance to type I PPB.

Heravi-Moussavi et al. conducted the first comprehensive analysis of *DICER1* in OSCSTs and identified a *DICER1* mutation in 60% of SLCTs, all of which were restricted to the RNase IIIb domain; in addition to a second germline mutation in a subset of cases; they also found *DICER1* mutations in 1 of 14 nonseminomatous testicular germ cell tumors, 2 of 5 ERMS, and in 1 of 266 epithelial ovarian and endometrial carcinomas^50^. Following this report, other studies have reported a similar incidence of *DICER1* mutation in SLCT, ranging from 32% to 98% of cases^44–48,51,53^. This broad range is partially explained by the enriched population bias for *DICER1* carriers in some of the studies. Additionally, intermediate or poorly differentiated SLCTs are almost universally associated with a *DICER1* mutation with a prevalence of 97% to 100% compared to well-differentiated cases of which only 12% in one study were associated with a *DICER1* mutation^47,48,53^. Given this strong association of *DICER1* mutation in SLCT, it is probably advisable for appropriate tissue testing especially in the younger age patients with moderately to poorly differentiated SLCTs with retiform or heterologous features. *DICER1*-associated SLCTs present at a younger age compared to the sporadic SLCT^44–47,51^. About 50% of *DICER1*-associated SLCTs are stage Ia and are free of disease after a median follow-up of 19 months in contrast to the sporadic tumors in which approximately 15% of patients had a recurrence^47^.

One particularly unusual example of a SLCT was one that presented in the lung as a cystic and solid mass in a 1-year-old male which was thought clinically to represent a PPB and was referred to the IPPBR (unpublished case). The tumor had pathogenic variant of *DICER1*. This case serves as an extraordinary example that *DICER1*-associated neoplasms are not restricted to the usual sites of presentation as in the case of PPB-like sarcomas arising in the kidney or peritoneum.

#### Gynandroblastoma

Gynandroblastoma is a sex-cord tumor with Sertoli-Leydig cell and an adult or juvenile granulosa cell pattern^39,49^, which predominantly occurs in the ovary with only the rare example in the testis^60^. The IOTSTR/IPPBR reported one case of gynandroblastoma in a patient with a germline *DICER1* mutation among 325 OSCSTs from 296 families with PPB^49^. Five additional cases in patients from 14- to 32-years of age (median age 16-years) have been reported subsequently by the IOTSTR^47^. In these cases, the SLCT component had intermediate differentiation and most cases were stage Ia. Four cases of the five were sequenced and all four had a *DICER1* RNase IIIb hotspot mutation; three of the four patients had a germline *DICER1* loss-of-function mutation^47^.

Another study evaluated a large cohort of gynandroblastomas consisting of 16 cases in patients from 14- to 80-years of age (median age 24.5-years); the granulosa cell tumor component in 10 cases had juvenile features with solid nodules of polygonal cells with or without luteinized features and follicular-like structures with granular basophilic secretions (62.5%)^61^. Only three cases had a *DICER1* hotspot mutation but importantly all of these cases had features of a moderate to poorly differentiated SLCT component with a juvenile granulosa cell component^61^. None of the cases had a *FOXL2* mutation. Of 26 gynandroblastomas tested for *DICER1*, ten (38%) had hotspot *DICER1* mutations within the RNase IIIb domain^47–49,61^. When a gynandroblastoma is encountered especially in the presence of an intermediate or poorly differentiated SLCT component, appropriate tissue testing and genetic counseling are indicated since *DICER1* carriers have an increased risk for the development of SLCT and gynandroblastoma. A pelvic ultrasound every 6 to 12 months is recommended until at least 40-years of age^35^.

### Embryonal rhabdomyosarcoma of the cervix (cERMS)

**Fig. 9:**
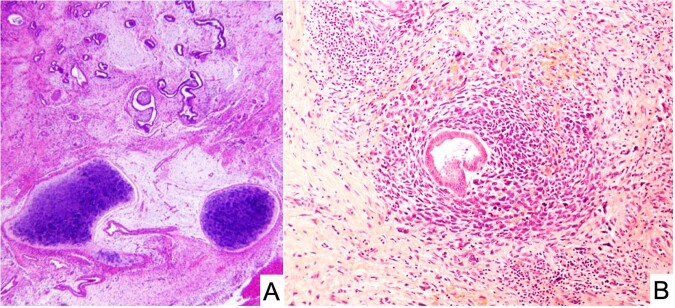
Embryonal rhabdomyosarcoma of the cervix. **A** A polypoid “vaginal” tumor presented in a 9-year-old female showing scattered endocervical glands with variably prominent subepithelial concentration of small cells and nodules of cartilage. PPB-type IR was also detected in the lung and a pathogenic heterozygous germline *DICER1* mutation was detected. **B** Endocervical gland encircled by embryonal rhabdomyosarcoma in an 11-year-old female with a cervical-vaginal mass. She subsequently developed a Sertoli-Leydig cell tumor at age 13 years and had a pathogenic heterozygous germline *DICER1* mutation.

One of the early reports of cERMS consisted of 13 cases in patients between 12- and 26-years of age who presented with vaginal bleeding^62^. In contrast to vaginal RMS with a median age at diagnosis of 2 years, cERMS is seen in older children, adolescents and young adults with a median age of 13–14 years^63^. However, a vaginal biopsy of an ERMS does not permit a distinction between a vaginal and cervical origin, but as noted the age at presentation as well as additional clinical studies may be helpful in the individual case. Uterine RMS presenting in the cervix of an adult occurs in younger age women than in those arising in the corpus^64^. The cERMS typically has the features of the favorable botryoid ERMS with a cambium layer and in almost 50% of the cases foci of cartilage are identified, a seemingly unique feature of ERMS within the *DICER1* setting unlike the absence of cartilage in the sporadic ERMS (Fig. 9A, B); this same combination of ERMS and cartilage is present in PPB as well as other *DICER1*-associated neoplasms. A noteworthy finding in the report of 13 cases was a prior history of SLCT in two patients; the authors stated that “the combination of a rare cervical tumor and a rare ovarian tumor in these two patients suggests more than a chance association”^62^. Subsequent reports have documented this association, one in a 27-year-old woman with a SLCT and a prior history of a cERMS at 14-years of age^65^, and another in a 13-year-old presenting with a synchronous SLCT and cERMS^66^.

The association of cERMS and SLCT was later reported in a cohort of 14 cases; these patients presented from 9-months to 32-years of age^67^. In addition to the botryoid pattern of ERMS, the hypocellular myxoid stroma in 6 cases (43%) contained one or more nodules of cartilage (Fig. 9A, B). One patient had a history of type IR PPB in early childhood and was found to have a germline *DICER1* mutation. Another patient had a history of type II PPB, with an unknown *DICER1* status and a third patient had a history of multinodular goiter and a SLCT^67^. Prior to the latter report, Foulkes et al identified four patients with cERMS, all of whom had a germline *DICER1* mutation^68^. Another case was subsequently reported in a 13-year-old female with cERMS and type IR PPB which was found to have a deleterious germline mutation in exon 12 as well as a somatic mutation^69^. Two additional cases of cERMS with a germline *DICER1* loss-of-function mutation have been reported^70^. These studies present the case that cERMS is a DICER-associated tumor. Molecular studies should be performed in the problematic case of an ERMS presenting as a vaginal mass.

Given the association of *DICER1* mutations with cERMS, de Kock and colleagues^71^ sought to evaluate the practical utility of *DICER1* testing as an aid in the challenging differential diagnosis of uterine adenosarcoma; these authors assessed 19 cERMS and 27 uterine adenosarcomas with a consensus diagnosis, and found that 18 cases (95%) of cERMS had a *DICER1* mutation compared to 7 cases (26%) of uterine adenosarcoma. Germline *DICER1* mutations were only identified in those with cERMS (6 of 12 cases tested) and in none of the adenosarcoma cases tested. Based on these results an absence rather than the presence of *DICER1* mutation could aid in this differential diagnosis, but a careful morphologic assessment of these tumors generally settles the diagnostic dilemma. A cERMS has an exclusive pattern of RMS whereas adenosarcoma is usually a low-grade spindle cell sarcoma.

ERMSs arising elsewhere in the genitourinary tract have been associated with *DICER1* syndrome; three cases with germline loss-of-function mutations have been described in the urinary bladder^70^, one case in the fallopian tube with a germline *DICER1* mutation, two cases in the ovary with somatic *DICER1* mutations^72^, and another case in the ovary with a germline *DICER1* mutation in exon 8 and a somatic mutation^73^. The ERMS in the fallopian tube and ovaries showed a cambium-like layer and nodules of mature cartilage; these histologic features overlap with PPB and cERMS. Unfortunately the ERMS of the urinary bladder did not have an available histologic description^70^. As with other suspected examples of *DICER1*-associated neoplasms, especially non-vaginal ERMS in the müllerian tract, appropriate molecular testing on the neoplasm should be pursued in addition to a discussion with the clinician^35,74^.

## Pediatric paratesticular sarcomas

Paratesticular neoplasms are those tumors arising from the testicular collecting system, the tunica and the spermatic cord; these tumors are uncommon and approximately 30% are malignant of which over 90% represent sarcomas with liposarcomas as the most common type^75,76^. Paratesticular sarcomas in children are principally ERMSs with or without a spindle cell pattern or spindle cell RMS in 75% or more of cases presenting in infancy or adolescence^77^. A *DICER1* mutation was identified in one low-grade “myxoid” sarcoma among 15 paratesticular sarcomas in children^78^; this tumor was composed of small cells embedded in a myxoid background with no increase in mitosis or anaplasia. There was an absence of rhabdomyoblastic or chondroid elements unlike several other examples of *DICER1*-associated extrapulmonary sarcomas. The presentation of a *DICER1*-associated neoplasm in the male reproductive tract contrasts at the moment with the apparent more common occurrence in females^18,79^.

It is recognized that a primary intraabdominal neoplasm may metastasize or directly extend from an abdominal or retroperitoneal location. Though we have not encountered a case as yet, a primary PPB-like peritoneal sarcoma presenting as a scrotal mass is a potential clinical scenario.

## The urinary system

Similar to the lung, where *DICER1* is essential for the branching morphogenesis of the epithelium^19,80,81^, the loss of *DICER1* expression in the developing kidney results in apoptosis in the progenitor nephron epithelium and premature termination of nephrogenesis and the development of renal cysts due to the apoptosis and loss of cell proliferation^20,80,81^. In addition, loss of *DICER1* function from the collecting duct epithelium is associated with hydronephrosis and collecting duct cysts^82^. In light of these findings in animal models, *DICER1* is considered essential for the survival of the nephron epithelium and differentiation of the ureteric bud epithelium. It is not entirely surprising that some patients with a PPB also had a cystic nephroma (CN) or a family history of CN^83^. The earlier paradigm was that CN in children was a representative of the Wilms tumor spectrum which was also thought to include the so-called adult CN/mixed epithelial and stromal tumor^84–86^. However, the latter neoplasm is regarded as a separate entity from pediatric CN and only rarely has a DICER mutation^87,88^.

Given the importance of *DICER1* in the development of the urinary system, a comprehensive evaluation of 89 individual *DICER1* carriers was compared to 61 family controls^89^. The presence of “renal cysts” was similar in both groups, 17% and 22% of known *DICER1* carriers and family controls, respectively with no differences in renal function between the two groups. Only the *DICER1* carriers had findings of nephrolithiasis or nephrocalcinosis which was present in 8 individuals (9%)^89^. To date, this is the most comprehensive study characterizing the spectrum of non-neoplastic renal abnormalities in *DICER1* carriers, and further studies with a larger population-based analysis are needed to further understand the full impact of germline *DICER1* mutations upon renal development and function.

### Cystic nephroma (CN) and anaplastic (DICER1) sarcoma of kidney (ASK)

**Fig. 10:**
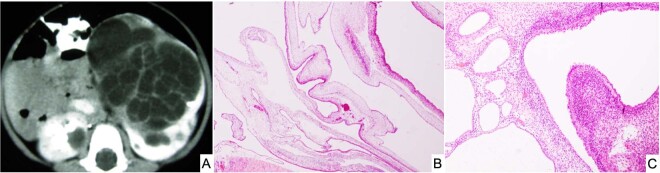
Cystic nephroma, pediatric type. **A** Circumscribed multicystic renal lesion in this CT image from a 2-year-old male who presented with an abdominal mass. A similar-appearing lesion on CT was present in the lung (PPB type I not shown). **B** Several delicate septal structures display architectural similarities to PPB type IR. **C** Multiple cysts in a fibrous stroma and one larger cyst with a hypercellular subepithelial mantle of spindle cells with a resemblance to PPB type I. Overtly sarcomatous elements were not present.

Pediatric CN (pCN) is a multiloculated cystic neoplasm presenting at or before 4-years of age as an unilateral, well-demarcated renal mass (Fig. 10A–C)^85,88^. Histologically, the cystic structures are lined by a simple epithelium with flat, cuboidal or hobnail features and the septa are composed of a bland fibrous stroma with scattered entrapped benign tubular structures, whose architectural features are similar to type I or IR PPB (Fig. 10B, C). The stroma is devoid of any immature nephroblastic elements which is the essential distinguishing histologic feature from the cystic partially differentiated nephroblastoma (CPDN)^84,88^.

In a report from the IPPBR, it was noted that there was a familial association between PPB and pCN in approximately 12% of cases; the pCN presented synchronously with the PPB in 10% of cases^90^. With the recognition of germline *DICER1* mutation in PPB^9^, the status of *DICER1* in 20 pCNs was evaluated and 70% of cases had an biallelic loss-of-function *DICER1* mutation, all of which were considered as a deleterious truncating mutations^91^. None of the CPDNs had a *DICER1* mutation to dichotomize these two tumors from each other. Unfortunately, germline testing was not performed in these cases but based upon our prior findings, 80% of *DICER1* loss-of-function mutations are germline in PPB^29^. In an earlier study by Bahubeshi et al., two families with familial CN were found to have a *DICER1* mutation in exon 23 in one family and in exon 25 in the other^92^. To date, the presence or absence of a *DICER1* mutation distinguishes a pCN from CPDN^87^.

**Fig. 11:**
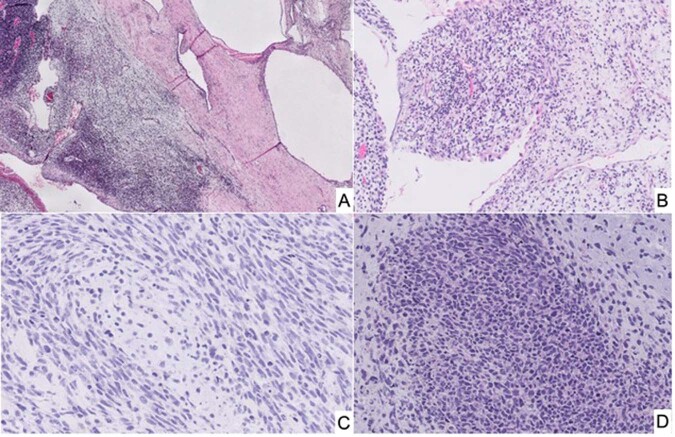
Anaplastic sarcoma of kidney (*DICER1* renal sarcoma) in the right kidney of a 4-year-old male. **A** The solid and cystic neoplasm showing a focus of residual pCN and adjacent cysts with surrounding hypercellular mantles of primitive small cells with rhabdomyoblastic differentiation (not shown) (inset, focus of hyperchromatic cells beneath a cyst). **B** A focus of primitive small, round and spindle-shaped cells projecting into a cyst with a subepithelial mantle of similar-appearing cells. **C** Malignant-appearing spindle cells surrounding a focus of neoplastic cartilage. **D** Focus of undifferentiated round cells with adjacent neoplastic cartilage. As in the case of PPB types II and III, anaplasia is not present in every ASK despite the implication of its appellation.

Given the potential of type I PPB to progress to a high grade multipatterned primitive sarcoma, the question was whether a similar phenomenon of tumor progression occurred in the case of pCN. Tumor progression in the pCN was extremely uncommon as documented by only four cases in the IPPBR experience, but there was considerable pathologic overlap between the sarcoma arising in pCN and the solid component of type II/III PPB^91^. These renal sarcomas have been designated previously as anaplastic sarcomas of the kidney (ASK) which like type II/III PPB may or may not have obvious anaplasia in every case (Fig. 11A–C)^93^. The cysts of the ASK are indistinguishable from pCN. Faria and Zerbini documented a predominantly cystic mass of the kidney with solid areas composed of rhabdomyoblastic and cartilaginous differentiation in a 26-month-old girl^94^; these authors commented upon the apparent sarcomatous transformation of a pCN and the pathologic similarities to PPB. A similar case in a 19-year-old male was composed of primitive mesenchyme in a myxoid background with accompanying cystic spaces^95^. Another case in a 4-year-old boy was a multilobulated renal mass composed of primitive small cells with rhabdomyoblastic differentiation with features similar to type II PPB^96^. In a review of 20 ASKs, the microscopic collage consisted of spindle cells merging with primitive mesenchymal cells, anaplasia and chondroid nodules in 16 of 20 cases and a cystic component in 7 cases; these tumors, as noted by the authors, were similar to types II/III PPB^93^. Despite the appreciation of the similarities of pCN and ASK to PPB type I and types II/III, *DICER1* testing was not available in these cases^93–97^. A later study from the IPPBR of four ASKs on which *DICER1* testing was performed on three, two had a *DICER1* mutation, one with an exon 14 nonsense mutation and a missense hotspot mutation, and the other had a somatic missense mutation^91^. More recently, nine cases of ASK were evaluated for *DICER1* and *TP53* mutations^98^; 8 cases had a somatic RNase IIIb *DICER1* mutation and overexpression of TP53 by IHC was seen in 6 of 9 cases, but only three cases had a *TP53* mutation^98^.

Given the association of pCN and *DICER1* carriers, and the rare progression to ASK-*DICER1* renal sarcoma, an abdominal ultrasound is recommended during infancy at the time a chest CT is done for PPB surveillance and every 6 to 12 months until at least 8-years of age. After this age, an annual ultrasound could be considered until 12-years of age^32^.

### Wilms tumor (WT)

WT is the most common primary renal neoplasm of childhood with 15% of cases associated with a familial predisposition syndrome including the linkage with mutations/deletions in *WT1* (11p13) and others that pre-dispose to WT^99–101^. Among 375 children with PPB and their families, only four cases (1%) of WT were identified; only one case developed in a child with a history of PPB whereas the other cases occurred in family members of those who had a PPB^91^. Prior to the documentation of these latter four cases, prior studies have analyzed the *DICER1* status in WT^5,92,102^. In one study of 50 sporadic WTs, none had a *DICER1* mutation^92^. Another study evaluated 243 WTs and detected only 1 case (0.4%) with a *DICER1* mutation; this same patient also developed bilateral OSLCT^5^. Wu et al examined 191 sporadic WTs and found five different somatic *DICER1* mutations in four patients; these same authors reported three patients with hereditary WT who were found to have germline *DICER1* mutations and an additional somatic mutation^102^. Although very uncommon WT can be associated with *DICER1* germline mutations whose pathogenetic role remains undefined at present.

## The gastrointestinal system

The gastrointestinal system is the site of involvement by several syndromes and *DICER1* tumor predisposition syndrome is no exception^103,104^.

### *DICER1*-associated cystic hepatic neoplasm

The association of *DICER1* mutation with hepatic manifestations was reported in two cases of “mesenchymal hamartoma” of the liver (MHL) in 26-month-old and 75-month-old boys; both children had heterozygous pathogenic *DICER1* variants and an accompanying somatic hot-spot RNase IIIb *DICER1* mutation, in one case, and a heterozygous in-frame germline deletion in the other, but a somatic mutation was not seen^105^.

**Fig. 12:**
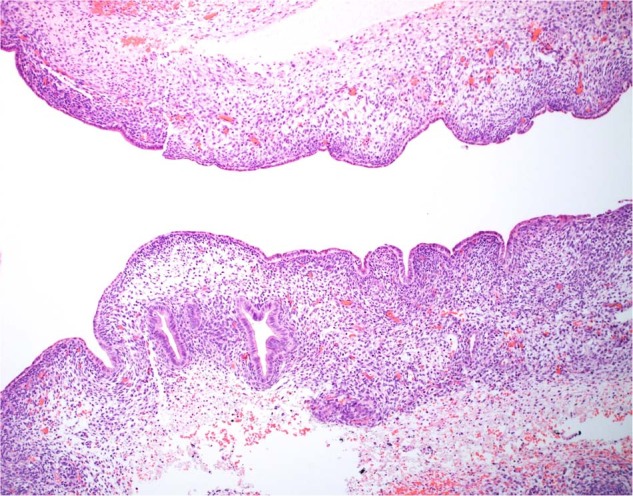
*DICER1* cystic hepatic neoplasm presenting as a cystic lesion of the liver in a 1-year-old male. A 10.7 cm mass with a multicystic cut surface showing one of multiple cysts lined by biliary type epithelium with a subepithelial zone of primitive small cells extending into the surrounding stroma. These cells demonstrated desmin immunopositivity. A pathogenic heterozygous germline mutation in *DICER1* was detected.

Dispute over the nature of these cystic tumors arose over their interpretation as MHLs since the latter infantile neoplasm has a well-documented activation of chromosome 19q microRNA cluster^106,107^. MHL is a cystic lesion which is characterized by a loosely cellular myxoid stroma with scattered, small dysplastic or malformed bile ducts resembling those of a bile duct plate abnormality and isolated islands of hepatocytes. Vargas and Perez-Atayde^108^ opined that the two cases of *DICER1*-associated MHL were not examples of classic MHL, but rather another type of cystic lesion of the liver. It could have been argued that both lesions of the liver had architectural similarities to type IR PPB or pCN. We have had an opportunity to study a case of a multicystic hepatic lesion from a 1-year-old infant who had a *DICER* germline mutation. The epithelial-lined cysts were surrounded by a cambium layer of rhabdomyoblasts and others with a concentric fibrous stroma; this multicystic lesion has the architectural and histologic features of type I PPB to conform with the familiar morphologic paradigm (Fig. 12). It is important to distinguish this novel cystic hepatic neoplasm with a *DICER1* mutation from classic MHL with its pathogenetic relationship to undifferentiated embryonal sarcoma liver^107,109^. More recently, a very similar cystic neoplasm of the liver to one described here has been reported whose pathologic features are similar to type I PPB with spindle cell sarcomatous progression in a young patient with a germline *DICER1* variant^110^.

### *DICER1*-associated hamartomatous polyps

Intestinal polyps are broadly classified into hamartomas and neoplastic/pre-neoplastic polyps. Hamartomatous polyps are benign proliferations of epithelial and mesenchymal elements presenting mainly in children and young adults. These polyps occur as either a sporadic lesion or in the setting of a polyposis syndrome with an increased risk for malignancy. The three types of hamartomatous polyps include juvenile polyps/juvenile polyposis syndrome, Peutz-Jeghers polyps of PJ syndrome and hamartomatous polyps in the *PTEN* hamartoma tumor syndrome^111–113^.

Though the reports of intestinal polyps in the *DICER1* setting are not detailed in terms of their pathology features, it appears that they are more aligned with hamartomatous types. Lallier M et al reported a case of bilateral PPBs and bowel obstruction due to multiple small intestinal polyps in a 5-week-old girl which was also documented in another study^90,114^. This patient also developed a cystic lesion in the kidney without pathologic confirmation, but likely represented a pCN in addition to another lesion in the iris which was thought to be metastatic PPB, but was more likely a ciliary body medulloepithelioma. At the time of publication^114^, the association with *DICER1* and PPB was still unknown but in retrospect, this child was manifesting the various *DICER1*-associated tumor types. The intestinal polyps in this child were interpreted at the time as “juvenile polyps” without a histologic description or illustration^114^. Other better documented cases of hamartomatous polyps in *DICER1* carriers include a 19-year-old female with a type I PPB, bilateral pCNs and an ileal intussusception due to a 3.2 cm “juvenile polyp”^90^. Other examples of intestinal polyps include a 2-year-old girl with an “hamartomatous esophageal polyp”^68^ and a 9-month-old girl with a type I PPB and a jejunal hamartomatous polyp^115^. Five children with PPB, pCN and small intestinal intussusception due to polyps and two children with PPB and small intestinal polyp have also been reported^116^. A thorough pathologic characterization of these various polyps has remained incomplete.

**Fig. 13:**
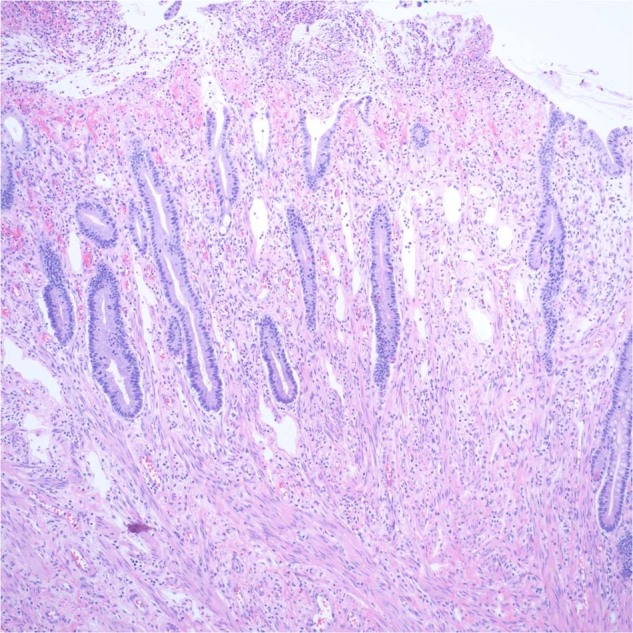
Hamartomatous polyp with juvenile polyp-like features. Small intestinal polyp in a child with PPB showing an ulcerated surface with elongated crypts and a lamina propria with bands of smooth muscle.

A previously unpublished case of a hamartomatous polyp in a patient with a history of PPB is presented here to provide a somewhat more detailed description of the pathologic features. The polyp had bands of smooth muscle haphazardly arranged through the lamina propria and extending to the surface without the arborizing and lobulated architecture of a PJP. There were no dilated crypts or inflammation of the lamina propria which are often present in the typical juvenile polyp (Fig. 13). In addition, the polyp had a prominent vascular pattern with dilated vessels highlighted with CD31 and CD34 in the intestinal villi, and a complex anastomosing dilated lymphatic spaces which extended into the stalk of the polyp, highlighted by a D2-40 immunostain.

## The peritoneum and retroperitoneum

Two recent additions to the spectrum of *DICER1*-associated tumors include the peritoneal sarcoma with the designation of PPB-like peritoneal sarcoma^117^, and a presacral malignant teratoid neoplasm^118^.

### Pleuropulmonary blastoma-like peritoneal sarcoma

**Fig. 14:**
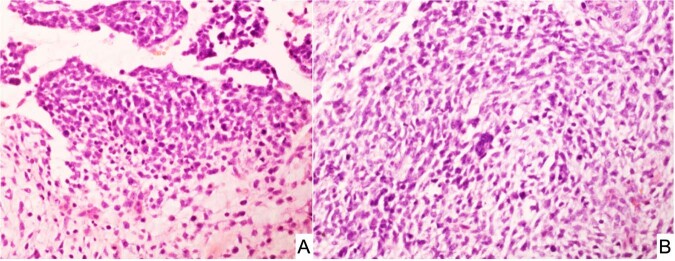
PPB-like peritoneal sarcoma arising in the pelvis of an adolescent female. **A** Cystic focus of the tumor demonstrating a hypercellular zone of primitive round cells with patchy myogenin positivity (not shown). **B** Solid areas of the tumor showing undifferentiated sarcoma with round and spindle cell features and an enlarged, anaplastic tumor cell.

Primitive sarcoma resembling PPB presenting in the peritoneal cavity has been reported in 7 children from 3 to 14 years with the fallopian tube (4 cases), pelvic sidewall (2 cases) and serosa of the colon as the apparent primary site^117^. None of these children had a past or contemporaneous history of PPB or other *DICER1–*associated neoplasms. The tumors all shared the morphologic spectrum of PPB including cystic spaces with underlying primitive small cells with or without rhabdomyoblastic differentiation (sarcoma botryoides-like pattern and nodules of cartilage similar to type I PPB; multilocular peritoneal cysts without underlying primitive mesenchymal cells resembling type IR PPB; and the remaining cases with cystic and solid features or a purely solid multipatterned primitive sarcoma of type II/III PPB (Fig. 14A, B). These similarities to PPB suggested a temporal progression from a simple multiloculated cyst to a solid multipatterned primitive sarcoma; however, this remains to be confirmed with the inclusion of additional cases. McCluggage and associates have reported three cases similar to our experience in addition to an ovarian ERMS^119^. Not only were there the overlapping morphologic features of PPB as in our series^117^, but biallelic loss of function RNase IIIb *DICER1* mutations were detected in six cases with available DNA and germline *DICER1* mutations were also present in four of the five patients^119^.

Prior to the characterization of PPB-like peritoneal sarcoma as a distinct entity, Warren et al reported two cases in 5-year old and 16-year old girls who presented with a right adnexal mass and a pelvic mass with omental and peritoneal metastases, respectively; these two cases as pointed out by the authors had pathologic features of types II/III PPB; both cases had a somatic *DICER1* mutation in addition to a germline mutation in one case which was the only one tested^79^. The authors also reviewed a total of 83 *DICER1*-associated sarcomas from the literature and concluded that there was a common morphologic motif with PPB of small primitive round cells, poorly differentiated spindle cells, primitive rhabdomyoblasts with a botryoid or nested pattern, chondroid nodules and anaplasia^79^. We would concur that any combination of these patterns or a predominance of one of these patterns should raise the possibility of a *DICER1*-associated neoplasm to initiate appropriate molecular testing, regardless of the site of presentation in a child or adolescent.

### *DICER1-*associated presacral malignant teratoid neoplasm

The most common sacrococcygeal neoplasm in the pediatric-aged population is the teratoma, representing over 90% of cases in this site^120^. Approximately 40–50% of all germ cell tumors in children present in the sacrococcygeal region^121^. Presacral teratomas are divisible into mature and immature types with malignancy usually represented by yolk sac tumor as multiple microscopic foci in those tumors presenting in infancy. More importantly to the best of our knowledge, the presence of ERMS in an infantile presacral teratoma should alert to the possibility that the tumor may represent a *DICER1*-associated neoplasm^120,121^. On the other hand, germ cell tumors of the testis in particular may give rise to a soft tissue sarcoma, including ERMS in less than 10% of cases in young adults^122^.

Two originally interpreted presacral immature teratomas presenting in a 1-week old boy and in a 4-month-old girl were found to have *DICER1* mutations in retrospect, specifically two nonsynonymous variants in one case, and a pathogenic germline *DICER1* mutation and a somatic hot-spot *DICER1* mutation in the other^118^. Both tumors had a distinct morphology composed of primitive neuroepithelial profiles and mesenchymal cells, spindle cells, and rhabdomyoblasts which were accompanied by nodules of primitive cartilage whose features should be familiar at this juncture with their similarities to other *DICER1*-associated tumors. It is important to be aware of this teratoid neoplasm because of its overlapping features with the immature sacrococcygeal teratoma. However, immature cartilage in association with spindle cells and rhabdomyoblasts should in the absence of other teratomatous elements and/or yolk sac tumor raise the possibility of *DICER1*-associated presacral malignant teratoid neoplasm.

## Head and neck

### Thyroid gland

Multinodular goiter (MNG) is the clinical designation for an enlarged, nodular thyroid without specificity as to the pathology, but with a differential diagnosis including thyroid carcinoma which is present in 5–15% of cases^123^. It is a manifestation in several syndromes and is common in areas with a high prevalence of iodine deficiency. In those regions with adequate iodine sources and MNG, it has been suggested that an underlying genetic susceptibility may exist, and two specific loci have been identified for familial MNG, one on chromosome 14q (*DICER1* on 14q32.13)^124^ and the other on Xp22^125^. Shortly after the identification of germline mutations in *DICER1* in PPB families, 5 families (53 individuals) with familial MNG were tested and three of the 5 families also had a familial history of OSLCT whereas the other two had only MNG^126^. Germline *DICER1* mutations were identified in 37 individuals showing a direct linkage of *DICER1* mutations and MNG, and notably familial MNG and OSLCT were independent of the occurrence of PPB^126^. A subsequent study identified 12 distinct *DICER1* hot spot mutations, all affecting the metal-iron binding residues in 10 patients with germline *DICER1* mutations^127^. Another study of 145 *DICER1* carriers and 135 family controls found a significantly higher cumulative incidence of MNG in the *DICER1* carriers independent of gender^128^. It has been shown that 3 of 4 and 1 of 6 *DICER1* carrier women and men develop MNG, respectively; MNG is estimated to have a 10–20% penetrance in *DICER1* carriers^128,129^. *DICER1* carriers have a 16- to 24-fold increased risk of developing thyroid carcinoma which is thought to be due to biallelic mutations in *DICER1* leading to an increase prevalence of benign thyroid nodules which over time acquire additional genetic alterations with malignant progression^128^. The latter theme is likely relevant to the tumor progression in type I PPB and pCN and other less well-studied *DICER1*-associated neoplasms.

### Differentiated thyroid carcinoma (DTC)

It is well established that exposure to radiation and high-dose chemotherapy is associated with an increased risk for the development for DTC^130,131^. Not surprisingly DTC has been reported in two children who received radiation therapy in the treatment of their PPBs^132,133^. At the time of those reports it was not clear whether the DTCs were a consequence of radiation exposure or a possible genetic linkage. A subsequent case of DTC was reported in a child with a history of PPB who developed recurrent disease and received salvage chemotherapy followed by high-dose chemotherapy and hematopoietic stem cell transplantation, but no radiation^134^.

A direct association between DTC and PPB, specifically involving *DICER1* mutations, was initially suggested in a report of three patients with a history of PPB; one developed MNG^129^, and was subsequently diagnosed with an invasive follicular variant papillary thyroid carcinoma (PTC)^134^. The second child had a type I PPB, developed a ciliary body medulloepithelioma at 6 years of age^135^, and one-year later was diagnosed with a follicular variant PTC; the third patient had a history of PPB and pCN and at 11.5 years was found to have a PTC^129^. All of these patients had a pathogenic germline *DICER1* mutation and an acquired somatic *DICER1* mutation affecting the RNase IIIb domain^123^. One caveat in these cases is that each patient received chemotherapy after the diagnosis of PPB and that the latter mutagenic event could explain the devolvement of DTC. However, a subsequent study reported a *DICER1* family with MNG; two patients in this kindred, 12- and 14-years old females, presented with MNG whose thyroidectomies showed a PTC; one of these patients subsequently developed a pCN and virilization secondary to an OSLCT^136^. Additionally, the mother and a sibling also developed DTC; this report served to document that DTC occurs in *DICER1* carriers without prior radiation and/or chemotherapy^136^.

Familial-syndromic PTC is reported in 10–14% of cases and in some of these cases, there is a germline mutation as in familial adenomatous polyposis, Cowden syndrome, Carney complex and *DICER1* syndrome^137,138^.

Follicular carcinoma (FC) in children is considerably less common than PTC, representing less than 2% of all DTCs in childhood; it is thought to be genetically different from FCs in adults with their *H/K/NRAS* variants and *PAX8-PPARy* fusions^139–141^. However, the incidence of these mutations has been largely unknown in the pediatric population until the study of 15 children with FC; a somatic *DICER1* mutation was present in 8 patients (53%) with mutated RNase IIIb domain in addition to germline mutations in four patients^142^. Coexistent nodular hyperplasia and follicular adenoma were significantly more frequent in *DICER1*-mutated FC compared to those without the mutation; another observation was that all cases of FC in children less than 10-years of age at diagnosis had a *DICER1* mutation^142^. Any newly diagnosed FC in a child under the age of 10 years should be evaluated for a *DICER1* mutation. In terms of prognosis none of the FCs in children showed evidence of recurrence over a median follow-up of 8.1 years but extensive capsular extension was only seen in cases with a *DICER1* mutation^142^.

FC and PTC in the pediatric population are clinically and genetically distinct from PTC in adults. Although associated with a high rate of regional lymph node metastasis, PTC in children has an excellent survival with a 30-year disease free survival of 99–100%^143^. In a study of 40 consecutive thyroidectomy specimens from children, *DICER1* mutations were identified in 3 (10%) of 30 PTC, two of 10 benign thyroid nodules were found to have a germline *DICER1* mutation and an additional somatic mutation within the RNase IIIb domain^144^.

Of note, *DICER1* mutations in DTC exclusive of *DICER1* carriers is rare with a somatic mutation frequency of approximately 0.6% (3 of 507 cases from the Cancer Genome Atlas Research Network [accessed on 12/15/2020]), and when present should raise concern about an undetected germline *DICER1* mutation^145^. However, as noted above in the pediatric population this frequency is significantly higher and thus a recommendation for screening for *DICER1* mutations in pediatric DTC is warranted. Given this increased risk for the development of DTC, it is recommended that a thyroid ultrasound around 8-years of age in *DICER1* carriers and then every 2 to 3 years, and in patients receiving chemotherapy, a baseline ultrasound is recommended and then annually for 5 years^32^.

Unlike many of the tumors associated with *DICER1* mutations, the pathology of the hyperplastic nodules and DTC has not been correlated with any specific histologic features which distinguishes them from the non-*DICER1* counterpart. It is important to question the possibility of a *DICER1* germline carrier in any case of nodular hyperplasia or DTC especially in a patient 40 years old or less. A more compelling case for a *DICER1* mutation is the child with a poorly-differentiated thyroid carcinoma which is discussed below.

#### Poorly-differentiated thyroid carcinoma

**Fig. 15:**
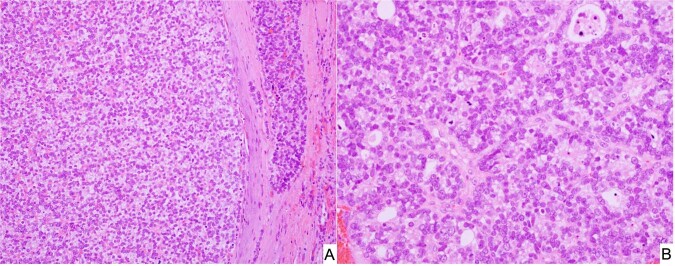
Poorly differentiated thyroid carcinoma in a 22-year-old female with a past history of Wilms tumor at age 7 years and a peritoneal sarcoma with cystic and rhabdomyosarcoma at age 11 years who was a DICER1 heterozygous germline variant. **A** The 3.5 cm, tan-pink, encapsulated mass showing tumor in the capsule and closely apposed small nests of uniforms tumor cells with dense nuclei and pale cytoplasm. **B** Trabecular and small follicular profiles of basophilic tumor cells demonstrating scattered mitotic figures.

Poorly-differentiated thyroid carcinoma (PDTC) is a rare neoplasm representing less than 1% of all thyroid malignancies and has a 60–70% 10-year survival^146,147^. PDTC in children is exceedingly rare and is usually documented as case reports before the implementation of the Turin criteria for the diagnosis of PDTC^147^. In a study by Chernock et al^148^, six cases of PDTC using the Turin criteria in patients less than 21-years old (age range: 14 to 19 years) were identified, of which 5 cases (83%) had somatic *DICER1* mutations, all encoding the metal-ion binding sites of RNase IIIb domain; these 5 cases had whole-exome sequencing and one germline pathogenic *DICER1* mutation and one loss of heterozygosity for *DICER1* were identified. Three of five patients with follow-up information died of disease, 8–24 months after diagnosis; all tumor-associated deaths were those cases with lymphovascular invasion, extrathyroidal invasion and a positive resection margin^148^. A recent case in a 22-year-old female with a *DICER1* germline variant and two previous DICER1-associated neoplasms showed extensive capsular and vascular invasion and multiple foci of tumor and nodular hyperplasia in the resected thyroid (Fig. 15).

### Cervical-thyroid teratoma

Teratomas arising in the thyroid and/or neck account for 1% or less of all extragonadal germ cell tumors; one of the larger case series consisted of 30 cases with a mean age of 12.4 years (range: newborn to 56-years)^149^. Those tumors in children, especially infants, were largely immature teratomas presenting at or soon after birth^150,151^. Unlike gonadal teratomas, these tumors lacked 12q alterations^152–154^. Rooper et al. reported that four malignant teratomas of the thyroid, unlike the mature and immature types, had somatic hotspot *DICER1* mutations^155^. Additionally, three of four non-malignant cases had non-hotspot *DICER1* mutations in the latter study, but germline mutations were not identified in these cases, likely representing sporadic mutations. All cases with hotspot *DICER1* mutations showed primitive and undifferentiated elements, spindle cells with rhabdomyoblastic differentiation and epithelial proliferation, and in three cases immature cartilage was also identified^155^; these cases have a resemblance to the previously discussed presacral malignant teratoid neoplasms. It is unclear at this time whether there is an association between *DICER1* mutated thyroid teratomas and *DICER1* predisposition tumor syndrome since to our knowledge no cases of cervicothyroidal teratomas have been reported in *DICER1* kindreds or in association with other *DICER1* related tumors in the experience of the IPPBR. Yet another primitive multipatterned neoplasm of the thyroid with *DICER1* alterations has been reported, apparently distinct from a teratoma, as a “malignant teratoid tumor” or “thyroblastoma” which is likely the same tumor type that Rooper and associates described^155,156^.

### Nasal chondromesenchymal hamartoma (NCMH)

**Fig. 16:**
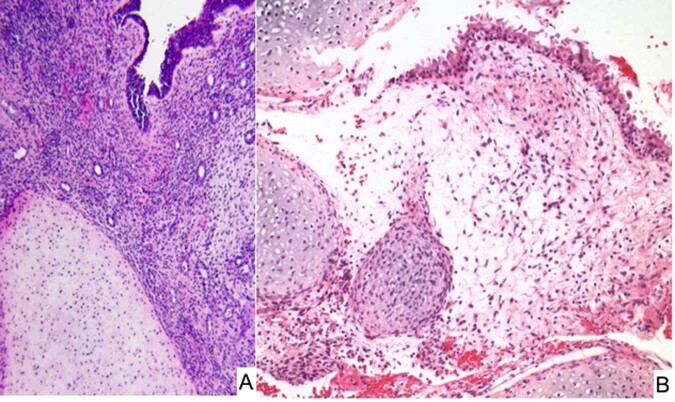
Nasal chondromesenchymal hamartoma. **A** This tumor demonstrating a well-differentiated nodule of cartilage with an accompanying spindle cell stroma without rhabdomyoblastic features. **B** Another example with a less cellular myxoid stroma and cartilaginous nodules with variation in differentiation.

NCMH is a benign polypoid mesenchymal tumor arising in the nasal cavity and/or paranasal sinuses whose microscopic features have some resemblance to the chest wall hamartoma of infancy^157^. The majority of cases in the original report presented in infants under 3 months of age (6 of 7 cases), but subsequent reports have documented a broader age range into adulthood^157–161^. A mass in the nasal cavity with extension to the paranasal sinuses, especially to the ethmoid sinus is the clinical presentation^157,161^. A cellular, somewhat immature mesenchyme without rhabdomyoblastic differentiation accompanies and surrounds irregular islands of mature appearing hyaline cartilage without atypical features; there is commonly a sharp interface between the chondroid nodules and the cellular to myxoid stroma (Fig. 16A, B). The myxoid stroma is composed of bland appearing slender spindle cells with hypo- or hypercellular features which may suggest RMS. In some cases, the more cellular stroma can have a storiform pattern^157,161^. Other features are osteoclast-like giant cell and blood-filled lake spaces with a resemblance to an aneurysmal bone cyst^147^.

There was a single child in the initial report with a history of PPB and it was suggested at the time that other neoplasms may develop in these patients before the known linkage with *DICER1*^157^. Almost ten-years later NCMH was reported in a 15-year-old girl with a clinical history of congenital phthisis bulbi, OSCST and PPB^162^. Subsequently, four cases of NCMH from approximately 625 children with PPB were identified from the IPPBR; this subset of cases ranged in age from 7 to 15 years^161^. Notably three of these children were part of the exploratory genetic project which established the initial associations of PPB with *DICER1* mutations^9^; nine additional cases from the IPPBR with a history of PPB subsequently developed a NCMH (age range: 6–27 years)^163^. Among 8 patients tested for a *DICER1* germline mutation, it was identified in 6, and in two a somatic *DICER1* missense mutation was detected in the NCMH providing compelling evidence of an association with the *DICER1* family of tumors.

In total the IPPBR has identified 13 cases of NCMH, 7 females and 6 males, with a mean age of 13 years (range: 6–27 years) which is substantially older than the mean age at presentation in the original report, 58 weeks (range: 5 days – 7 years) (*p* < 0.001). Of note the patient in the original series with a history of PPB was 7-years old^157^.

A thought-provoking observation is the description in the original report of case #7 of a predominant polypoid mass composed almost exclusively of islands of cartilage and a loose myxoid stroma with an epithelial lined cyst^157^. In the subsequent IPPBR case series reporting the association with *DICER1*, all cases had variably-sized cysts lined by respiratory epithelium^161^. This morphologic association of cystic structures with a myxoid stroma and accompanying islands of cartilage follows the histologic motif of the other *DICER1*-associated neoplasms. To date, there have been no observed examples of sarcomatous progression of *DICER1*-associated NCMHs though these tumors may locally recur and directly invade into the cranial space^157^.

## Central nervous system and eye

The central nervous system including the eye as a direct appendage of the brain is the site of primary *DICER1*-associated tumors while the brain is the most common metastatic site of PPB^164^. Primary *DICER1*-associated neoplasms affecting the central nervous system and eye include the following tumor types: ciliary body medulloepithelioma (CBME), pituitary blastoma, pineoblastoma, primary *DICER1*-associated sarcoma and embryonal tumor with multilayered rosettes. Kock et al. have provided a comprehensive review of the *DICER1* CNS manifestations^165^.

### Eye

Kaneko et al. demonstrated that *DICER1* levels are decreased in the retinal pigmented epithelium (RPE) in cases of extensive age-related macular degeneration, and showed in *DICER1* knockdown models that an increased accumulation of *Alu* RNA in RPE cells resulted in RPE degeneration^166^. Given this direct causality of *DICER1* and blindness, it was questioned arose whether individuals with germline *DICER1* mutations are predisposed to visual loss; this inquiry was addressed in a comprehensive family-based study in 103 patients with a pathogenic germline *DICER1* mutation and 69 family control subjects^167^. All subjects underwent an ophthalmic evaluation, with a mean age of 27 years and 37.9 years for the *DICER1* and control groups, respectively. Ocular abnormalities were more commonly seen in the *DICER1* group (22% vs 6%, *p* = 0.005) with the most common alterations involving the optic nerve, retinal pigmentary abnormalities, macular degeneration and an epiretinal membrane^167^. Regarding the visual acuity, most of those in the *DICER1* group had an acuity greater than 20/40 in both eyes. Notably during the study evaluation, ciliary body medulloepithelioma was identified in patients in the *DICER1* group within 1 year of the dilated eye examination. A recommendation has been made for an annual dilated ophthalmic examination in *DICER1* carriers, especially in those patients under the age of 10 years^167^.

#### Ciliary body medulloepithelioma (CBME)

CBME is the most common congenital and early childhood tumor of the nonpigmented epithelium of the ciliary body^168,169^. This primitive neuroepithelial neoplasm has been classified into nonteratoid and teratoid types, which is based on the absence or presence of heterologous elements including cartilage, rhabdomyoblasts and neuroglia^169–172^. Cystic or multicystic component is recognized in both the teratoid and non-teratoid CBME; both types behave in a benign or malignant manner correlating with the presence of poorly differentiated neuroepithelial profiles with or without rosettes, chondrosarcomatous and/or rhabdomyosarcomatous foci, and invasion of the uvea, cornea or sclera with or without extrascleral extension^168,170,172^. This composite of histologic features is another example of the common morphologic motif of several other pulmonary and extrapulmonary *DICER1*-associated neoplasms.

Four cases of CBME were identified among 299 enrolled PPB cases; three occurred in patients with PPB and another in the father of children with PPBs^135^. This observation was followed by a case report of 9-year-old girl with a CBME and history of PPB^173^. Additionally, Kaliki et al. reported a history of PPB in 2 patients among 41 cases of CBME^168^. Although there is a clear association of this rare ocular neoplasm with PPB and with *DICER1* syndrome, only one patient in these reports had germline and a somatic *DICER1* testing performed with a paternally inherited germline *DICER1* mutation^135^. The other study to our knowledge with genetic testing was an 18-year-old woman who presented with a 2-month history of painless visual loss and was found to have a 6 mm ciliary body mass; the tumor was characterized by a proliferation of neurotubular structures and cords surrounded by a loose stroma without heterotopic features and a somatic *DICER1* mutation was identified in this case in exon 26 (D1709N) without a germline mutation^174^.

### Brain

#### Pituitary blastoma

Pituitary blastoma is a neoplasm presenting in children under 2-years of age in the pituitary gland whose histologic features include primitive blastemal cells, glandular-like structures composed of small cells resembling Rathke epithelium and large secretory cells^175,176^. A total of 14 cases to date have been evaluated for *DICER1* mutations, and 11 (79%) have pathogenic heterozygous germline *DICER1* mutations and in the remaining three without a germline mutation, a somatic *DICER1* mutation involving the RNase IIIb domain was identified^165,177^. The diagnosis of a pituitary blastoma is an indication for germline *DICER1* testing.

#### Pineoblastoma

Pineoblastoma, another rare primitive neoplasm of the CNS, typically presenting in children to young adults; this tumor has been associated with germline *RB1* mutations in the setting of the so-called trilateral retinoblastoma^178^. This high-grade hypercellular neoplasm is composed of primitive small cells with occasional rosettes; the histologic pattern is shared by other primitive tumors in the CNS such as medulloblastoma and atypical teratoid/rhabdoid tumor so that location and imaging findings are important considerations in the diagnosis. When these tumors occur in those with germline *DICER1* mutations, there is commonly loss of heterozygosity of the wild-type *DICER1* allele which is contrary to the other *DICER1*-associated neoplasms in which the “second-hits” are somatic missense RNase IIIb hotspot mutations^165,179,180^.

### Primary *DICER1-*associated central nervous system sarcoma

Several examples of primary CNS neoplasms have been reported with histologic features similar to other *DICER1*-associated neoplasms including PPB^79,165,181–185^. One large case series consisted of 22 intracranial sarcomas of which 21 (95%) had *DICER1* hotspot mutations as well as *TP53* mutations in 50% of cases; germline testing was only performed on five cases of which two had a germline *DICER1* mutation^183^. Most of these tumors were supratentorial in location with only two infratentorially examples, one in the cerebellopontine angle and the other in the cerebellum. Another cohort of these tumors was reported by Kamihara et al; six patients from 3- to 15-years of age presented with a supratentorial tumor, all of which had one inactivating *DICER1* mutation and one hotspot mutation in the RNase IIIb domain^182^.

Many of these tumors have had a morphologic resemblance to type II/III PPB with areas ranging from solid to cystic foci and primitive malignant spindle cells with diffuse anaplasia, focal rhabdomyoblastic differentiation, primitive embryonal-type foci in some cases and less commonly chondroid differentiation. Given the overlapping features with PPB in a neoplasm in the CNS, a review of the imaging, including the chest, is necessary to rule out the possibility of metastatic PPB; however, most primary *DICER1*-associated sarcomas are located supratentorial whereas metastatic PPB tends to occur in the cerebellum^25^. We have seen metastatic PPB to the CNS develop shortly after the diagnosis of PPB, but have not seen a child with a PPB initially present with a brain metastasis.

### Embryonal tumor with multilayered rosettes (ETMR)-like cerebellar tumor

Two cerebellar tumors have been seen in an 8- and 11-month old girls; both tumors had features of an embryonal type neoplasm with multilayered rosettes with abundant neuropil and true rosettes; one of the cases in the 8-month-old girl additionally had chondroid differentiation^186^. Both tumors had somatic hotspot *DICER1* mutations, in addition to a germline *DICER1* nonsense pathogenic mutation. These two cases again highlight the importance of the potential histologic clues to a *DICER1*-associated neoplasm. The authors advised *DICER1* testing in any embryonal or primitive appearing CNS tumor, not otherwise specified^186^. An additional case presenting in a 2-month-old girl has been reported; this tumor was composed of primitive small cells in a background of neuropil and scattered multilayered rosettes and pseudo-rosettes, in addition to rare rhabdomyoblasts^165^. A primitive CNS tumor, especially with rhabdomyoblastic and/or chondroid differentiation, should prompt *DICER1* testing.

## Other non-neoplastic *DICER1* associations

### Macrocephaly

Macrocephaly is defined as a head circumference greater than 97^th^ percentile compared to the published general population. In a cohort of 67 *DICER1* carriers, 28 (42%) qualified as macrocephalic and none had an occipital-frontal circumference below the 3^rd^ percentile which was significantly higher when compared to a cohort of 43 family controls of which only 12% were macrocephalic^187^. Additionally, this study revealed that *DICER1* carriers were taller than family controls after controlling for gender. These non-neoplastic overgrowth manifestations in combination with other findings as discussed in this review could facilitate the identification of suspected *DICER1* carriers.

### Dental abnormalities

Prior studies have shown that knockout *DICER1* models results in various tooth abnormalities since miRNAs have a crucial role in tooth epithelial stem cell differentiation^188–190^. It was then hypothesized that *DICER1* carriers may present with a spectrum of dental abnormalities and was tested in 57 *DICER1* carriers and 55 family controls^191^. Some significant differences included an excess of crown bulbousness, taurodontism and periodontitis in the *DICER1* carriers^188^. Although not statistically significant, supernumerary teeth, enamel defects and abnormal molar morphology were more common in the *DICER1* carriers.

### Global developmental delay, lung cyst, overgrowth and Wilms tumor (GLOW syndrome)

GLOW is an acronym for global developmental delay, lung cyst, overgrowth and Wilms tumor as a syndrome association^192^. Two patients, a 9-month-old boy and a 14-month-old boy, had development milestone delay, height and weight greater than 75^th^ percentile, head circumference greater than 98^th^ percentile, nephromegaly with Wilms tumor, lung cysts and dysmorphic features including hypertelorism, flat nasal bridge and frontal bossing. A heterozygous *DICER1* de-novo mutation was identified in one case, and a *DICER1* missense mutation in the other child; these mutations were present in varying frequencies in the available tissues for testing. These findings suggest that in addition to its oncogenic role, *DICER1* has any number of other gene functions including growth signaling pathways as in the case of *DICER1*-associated macrocephaly.

## Commentary/conclusion

Cancer predisposition syndromes (CPS) with their germline mutations have come to occupy a increasingly central role in our understanding in the development of tumors in all age groups. One of the earliest CPS was recognized in the setting of retinoblastoma which served as the basis of the Knudson two-hit hypothesis of the tumor suppressor gene with biallelic loss of RBI gene^193,194^. With the loss of both copies, additional genetic and epigenomic events facilitated tumor progression of retinoblastoma^195^. Several of these CPS were well-documented in children before whole exonic and genomic sequencing became available through classic kindred analysis as in the cases of neurofibromatosis types I and 2, familial adenomatous polyposis, Li-Fraumeni syndrome (LFS) and multiple endocrine neoplasia types 1 and 2. It is currently estimated that 10% of solid tumors in children are manifestations of a recognized CPS; however, one study from Denmark concluded that the frequency of a germline pathogenic mutations may approach 50%^196^.

*DICER1* syndrome (*DICER1* tumor predisposition syndrome) is one of more recently recognized CPS in childhood whose germline mutation, a heterozygous *DICER1* mutation, was first recognized just over 16 years ago^9^. The initial event in the evolution of the *DICER1* syndrome was the report of a seemingly unique, as yet not fully characterized primary neoplasm of the lung and/or chest wall in children, which was designated a PPB to differentiate it from the adult pulmonary blastoma^16^. After first report, subsequent cases pointed to a possible familial predisposition and that other extrapulmonary neoplasms occurred in children with PPB and within affected kindreds^17^. A solid, multipatterned primitive sarcoma was the characteristic pathologic feature of this neoplasm from the report of the first 11 cases^16^, but it subsequently became apparent that this solid neoplasm was the ultimate stage in a lesion which began as a circumscribed, multiloculated cyst in the lung which was regarded at one time as a type 4 congenital pulmonary airway (adenomatoid) malformation. Beneath the epithelial lining of the cysts in many of these lesions, primitive small cell population with and without rhabdomyoblastic differentiation and small nodules of immature cartilage were present in the reconceptualized type I PPB^23^. Proliferation of these neoplastic cells eventuated in progressive overgrowth of the cysts with the formation of a mass with or without residual cystic foci (type II or type III PPB). This evolution from a multiloculated cyst to a solid tumor with the pathologic features of the first 11 cases was correlated with the median age at diagnosis (cystic or type I PPB at 8 months and the solid type III PPB at 41 months), and a 5-year OS of 91% to 53% with progression to the solid, aggressive type III PPB^25^. These observations were the basis of the argument that the PPB underwent tumor progression from a cystic lesion to a solid high grade neoplasm with its prognostic consequences.

CPS are defined by their canonical germline mutations, but also by the phenotype(s) of their associated neoplasms^197^. Each of the mutated genes with the end result of a clinical malignancy of one type or another has set molecular perturbations such as disordered genomic stability and cell cycle dysregulation as in the case of LFS [TP53] or miRNA processing in the *DICER1* syndrome^198,199^. In the *DICER1* syndrome, the spectrum of tumor types has expanded as well as our understanding of *DICER1* in tumorigenesis (Table 1). It is the clinical and pathologic manifestations of the *DICER1* syndrome which is the focus of this review. It was appreciated in the early stage of our studies that PPB was probably not the sole manifestation of an as undefined syndrome.

In addition to a discussion of the various *DICER1*-associated tumors, the other point of emphasis is the morphologic similarity of several of these neoplasms which reflects a common pathogenetic and possibly histogenetic pathway whose pathologic features overlap with those of PPB (Table 1). Some of these tumor are predominantly cystic in their early stage of evolution, possibly as a manifestation of defective branching morphogenesis, a key developmental role of *DICER1*^19,21^. Both the lung and kidney rely upon branching morphogenesis for normal development^19–21^. In fact, normal development, ostensibly normal with one germline copy of non-mutated *DICER1*, sets the stage for the potential consequences with the somatic loss of the second copy in the two-hit model^200^. In the case of the tumorigenesis pathway, the second somatic hit and its particular anatomic site determine where the primary *DICER1*-associated neoplasm(s) presents in one or more organs or elsewhere. Additional mutations, such as in TP53 and other as yet unidentified genomic or epigenomic events^32^, facilitate the progression to a *DICER1*-associated neoplasm with one or multiple morphologic patterns in a complex collage of rhabdomyoblasts with features of ERMS, spindle cells, blastemal islands with or without rhabdomyoblastic differentiation, nodules of cartilage with fetal or immature or sarcomatous features and a population of larger, pleomorphic cells with or without anaplasia in selected tumor types. Another feature is the presence of immature tubules resembling primitive tubules with neural or nephrogenic features are seen in those *DICER1*-associated neoplasms with teratoid features which together with the other differentiated sarcomatous components may suggest or lead to the diagnosis of a germ cell neoplasm; these latter findings have been seen in the eye, thyroid, cervix, central nervous system and pre-sacral region. There is a set of pathologic features in the various *DICER1* neoplasms that should set in motion a consideration whether the patient may have a *DICER1* heterozygous germline mutation.

As a final note, there are some interesting parallels between the *DICER1* tumor syndrome and LFS as compared to some of the other inherited tumor predisposition syndromes whose tumor types are confined to a limited number of sites and tumor types. LFS is characterized by five core tumor types accounting for 75% of all neoplasms including carcinoma of the breast (25–30% of cases), soft tissue sarcomas (~15%), brain tumors (13–15%), adrenocortical carcinoma (10%) and osteosarcoma (10%). Likewise, the tumors of the *DICER1* tumor syndrome are found in a number of organ systems as discussed in this overview and are as diverse as the pineoblastoma to the PDTC, but unlike LFS there is some unity to the morphologic pattern which is predominantly sarcomatous and centered around ERMS with a cambium layer of rhabdomyoblasts beneath an epithelia appropriate to the anatomic site or a more diffuse solid pattern, often intermixed with non-RMS areas, cartilage and anaplasia; these features in some combination are maintained to some extent throughout the spectrum of *DICER1*-associated neoplasms with a few exception.

## References

[1] Denli AM, Tops BB, Plasterk RH, Ketting RF, Hannon GJ (2004). Processing of primary microRNAs by the Microprocessor complex. Nature.

[2] Zhang H, Kolb FA, Jaskiewicz L, Westhof E, Filipowicz W (2004). Single processing center models for human Dicer and bacterial RNase III. Cell.

[3] Bernstein E, Caudy AA, Hammond SM, Hannon GJ (2001). Role for a bidentate ribonuclease in the initiation step of RNA interference. Nature.

[4] Foulkes WD, Priest JR, Duchaine TF (2014). DICER1: mutations, microRNAs and mechanisms. Nat. Rev. Cancer.

[5] Slade I (2011). DICER1 syndrome: clarifying the diagnosis, clinical features and management implications of a pleiotropic tumour predisposition syndrome. J. Med. Genet..

[6] Wienholds E, Koudijs MJ, van Eeden FJ, Cuppen E, Plasterk RH (2003). The microRNA-producing enzyme Dicer1 is essential for zebrafish development. Nat. Genet..

[7] Bernstein E (2003). Dicer is essential for mouse development. Nat. Genet..

[8] Kumar MS (2009). Dicer1 functions as a haploinsufficient tumor suppressor. Genes Dev..

[9] Hill DA (2009). DICER1 mutations in familial pleuropulmonary blastoma. Science.

[10] Brenneman M (2015). Temporal order of RNase IIIb and loss-of-function mutations during development determines phenotype in pleuropulmonary blastoma/DICER1 syndrome: a unique variant of the two-hit tumor suppression model. F1000Res.

[11] de Kock L, Wu MK, Foulkes WD (2019). Ten years of DICER1 mutations: provenance, distribution, and associated phenotypes. Hum. Mutat..

[12] Kim J, Field A, Schultz KAP, Hill DA, Stewart DR (2017). The prevalence of DICER1 pathogenic variation in population databases. Int. J. Cancer.

[13] Robertson, J. C., Jorcyk, C. L. & Oxford, J. T. DICER1 syndrome: DICER1 mutations in rare cancers. *Cancers* **10**, 143 (2018). 10.3390/cancers10050143 PMC5977116 29762508

[14] Kim J, Schultz KAP, Hill DA, Stewart DR (2019). The prevalence of germline DICER1 pathogenic variation in cancer populations. Mol. Genet. Genom. Med..

[15] Anglesio MS (2013). Cancer-associated somatic DICER1 hotspot mutations cause defective miRNA processing and reverse-strand expression bias to predominantly mature 3p strands through loss of 5p strand cleavage. J. Pathol..

[16] Manivel JC (1988). Pleuropulmonary blastoma. The so-called pulmonary blastoma of childhood. Cancer.

[17] Priest JR (1996). Pleuropulmonary blastoma: a marker for familial disease. J. Pediatr..

[18] Schultz, K. A. P. et al. DICER1 tumor predisposition. In *GeneReviews((R))* (eds. Adam, M. P. et al.) (Seattle, WA, 1993).

[19] Goodwin, K. & Nelson, C. M. Branching morphogenesis. *Development* **147** dev184499 (2020). 10.1242/dev.184499 32444428

[20] Costantini F, Kopan R (2010). Patterning a complex organ: branching morphogenesis and nephron segmentation in kidney development. Dev. Cell.

[21] Harris KS, Zhang Z, McManus MT, Harfe BD, Sun X (2006). Dicer function is essential for lung epithelium morphogenesis. Proc. Natl Acad. Sci. USA.

[22] Dehner LP (1994). Pleuropulmonary blastoma is the pulmonary blastoma of childhood. Semin. Diagn. Pathol..

[23] Priest JR (1997). Pleuropulmonary blastoma: a clinicopathologic study of 50 cases. Cancer.

[24] Hill DA (2008). Type I pleuropulmonary blastoma: pathology and biology study of 51 cases from the international pleuropulmonary blastoma registry. Am. J. Surg. Pathol..

[25] Messinger YH (2015). Pleuropulmonary blastoma: a report on 350 central pathology-confirmed pleuropulmonary blastoma cases by the International Pleuropulmonary Blastoma Registry. Cancer.

[26] Cummings NM (2010). Cystic primary pulmonary synovial sarcoma presenting as recurrent pneumothorax: report of 4 cases. Am. J. Surg. Pathol..

[27] Moog U, Felbor U, Has C, Zirn B (2020). Disorders caused by genetic mosaicism. Dtsch. Arztebl. Int..

[28] Guedes LB (2017). Analytic, preanalytic and clinical validation of p53 IHC for detection of TP53 missense mutation in prostate cancer. Clin. Cancer Res..

[29] Pugh TJ (2014). Exome sequencing of pleuropulmonary blastoma reveals frequent biallelic loss of TP53 and two hits in DICER1 resulting in retention of 5p-derived miRNA hairpin loop sequences. Oncogene.

[30] Seki M (2014). Biallelic DICER1 mutations in sporadic pleuropulmonary blastoma. Cancer Res..

[31] Vargas SO (2006). Cytogenetic and p53 profiles in congenital cystic adenomatoid malformation: insights into its relationship with pleuropulmonary blastoma. Pediatr. Dev. Pathol..

[32] Gonzalez IA (2021). Expression of p53 is significantly associated with recurrence-free survival and overall survival in pleuropulmonary blastoma (PPB): a report from the International Pleuropulmonary Blastoma/DICER1 Registry. Mod. Pathol..

[33] Murray MJ (2014). Serum levels of mature microRNAs in DICER1-mutated pleuropulmonary blastoma. Oncogenesis.

[34] Schultz KAP (2014). Judicious DICER1 testing and surveillance imaging facilitates early diagnosis and cure of pleuropulmonary blastoma. Pediatr. Blood Cancer.

[35] Schultz KAP (2018). DICER1 and associated conditions: identification of at-risk individuals and recommended surveillance strategies. Clin. Cancer Res..

[36] Merideth MA (2020). Gynecologic and reproductive health in patients with pathogenic germline variants in DICER1. Gynecol. Oncol..

[37] Solarski M (2018). DICER1 gene mutations in endocrine tumors. Endocr. Relat. Cancer.

[38] Hanley KZ, Mosunjac MB (2019). Practical review of ovarian sex cord-stromal tumors. Surg. Pathol. Clin..

[39] Schultz KAP (2016). Ovarian sex cord-stromal tumors. J. Oncol. Pr..

[40] Schneider DT (2003). Ovarian sex cord–stromal tumors in children and adolescents. J. Clin. Oncol..

[41] Meserve EEK, Nucci MR (2016). Peutz-Jeghers syndrome: pathobiology, pathologic manifestations and suggestion for recommending genetic testing in pathology reports. Surg. Pathol. Clin..

[42] Fuller PJ, Leung D, Chu S (2017). Genetics and genomics of ovarian sex cord-stromal tumors. Clin. Genet..

[43] Young RH, Welch WR, Dickersin GR, Scully RE (1982). Ovarian sex cord tumor with annular tubules. Review of 74 cases including 27 with Peutz-Jeghers syndrome and four with adenoma malignum of the cervix. Cancer.

[44] Kato N (2017). DICER1 hotspot mutations in ovarian Sertoli-Leydig cell tumors: a potential association with androgenic effects. Hum. Pathol..

[45] Karnezis AN (2019). DICER1 and FOXL2 mutation status correlates with clinicopathologic features in ovarian Sertoli-Leydig cell tumors. Am. J. Surg. Pathol..

[46] Goulvent T (2015). DICER1 and FOXL2 mutations in ovarian sex cord-stromal tumours: a GINECO group study. Histopathology.

[47] Schultz KAP (2017). DICER1-related sertoli-leydig cell tumor and gynandroblastoma: clinical and genetic findings from the International Ovarian and Testicular Stromal Tumor Registry. Gynecol. Oncol..

[48] Conlon N (2015). A survey of DICER1 hotspot mutations in ovarian and testicular sex cord-stromal tumors. Mod. Pathol..

[49] Schultz KAP (2011). Ovarian sex cord-stromal tumors, pleuropulmonary blastoma and DICER1 mutations: a report from the International Pleuropulmonary Blastoma Registry. Gynecol. Oncol..

[50] Heravi-Moussavi A (2012). Recurrent somatic DICER1 mutations in nonepithelial ovarian cancers. N. Engl. J. Med..

[51] Witkowski L (2013). DICER1 hotspot mutations in non-epithelial gonadal tumours. Br. J. Cancer.

[52] Wang Y (2015). The oncogenic roles of DICER1 RNase IIIb domain mutations in ovarian Sertoli-Leydig cell tumors. Neoplasia.

[53] de Kock L (2017). DICER1 mutations are consistently present in moderately and poorly differentiated Sertoli-Leydig cell tumors. Am. J. Surg. Pathol..

[54] Young RH, Scully RE (1985). Ovarian Sertoli-Leydig cell tumors. A clinicopathological analysis of 207 cases. Am. J. Surg. Pathol..

[55] Zaloudek C, Norris HJ (1984). Sertoli—Leydig tumors of the ovary A clinicopathologic study of 64 intermediate and poorly differentiated neoplasms. Am. J. Surg. Pathol..

[56] O’Brien PK, Wilansky DL (1981). Familial thyroid nodulation and arrhenoblastoma. Am. J. Clin. Pathol..

[57] Jensen RD, Norris HJ, Fraumeni JF (1974). Familial arrhenoblastoma and thyroid adenoma. Cancer.

[58] Benfield GFA, Tapper-Jones L, Stout TV (1982). Androblastoma and raised serum alpha-fetoprotein with familial multinodular goitre. Case report. Br. J. Obstet. Gynaecol..

[59] Prat J, Young RH, Scully RE (1982). Ovarian Sertoli-Leydig cell tumors with heterologous elements. II. Cartilage and skeletal muscle: a clinicopathologic analysis of twelve cases. Cancer.

[60] Takeda A, Watanabe K, Hayashi S, Imoto S, Nakamura H (2017). Gynandroblastoma with a juvenile granulosa cell component in an adolescent: case report and literature review. J. Pediatr. Adolesc. Gynecol..

[61] Wang Y (2018). DICER1 hot-spot mutations in ovarian gynandroblastoma. Histopathology.

[62] Daya DA, Scully RE (1988). Sarcoma botryoides of the uterine cervix in young women: a clinicopathological study of 13 cases. Gynecol. Oncol..

[63] Minard-Colin V (2018). Localized vaginal/uterine rhabdomyosarcoma-results of a pooled analysis from four international cooperative groups. Pediatr. Blood Cancer.

[64] Pinto A (2018). Uterine rhabdomyosarcoma in adults. Hum. Pathol..

[65] Golbang P, Khan A, Scurry J, Macisaac I, Planner R (1997). Cervical sarcoma botryoides and ovarian Sertoli–Leydig cell tumor. Gynecol. Oncol..

[66] McClean GE, Kurian S, Walter N, Kekre A, McCluggage WG (2007). Cervical embryonal rhabdomyosarcoma and ovarian Sertoli-Leydig cell tumour: a more than coincidental association of two rare neoplasms?. J. Clin. Pathol..

[67] Dehner LP, Jarzembowski JA, Hill DA (2012). Embryonal rhabdomyosarcoma of the uterine cervix: a report of 14 cases and a discussion of its unusual clinicopathological associations. Mod. Pathol..

[68] Foulkes WD (2011). Extending the phenotypes associated with DICER1 mutations. Hum. Mutat..

[69] Tomiak E, de Kock L, Grynspan D, Ramphal R, Foulkes WD (2013). DICER1 mutations in an adolescent with cervical embryonal rhabdomyosarcoma (cERMS). Pediatr. Blood Cancer.

[70] Doros L (2012). DICER1 mutations in embryonal rhabdomyosarcomas from children with and without familial PPB-tumor predisposition syndrome. Pediatr. Blood Cancer.

[71] de Kock L (2020). Significantly greater prevalence of DICER1 alterations in uterine embryonal rhabdomyosarcoma compared to adenosarcoma. Mod. Pathol..

[72] McCluggage WG (2020). Embryonal rhabdomyosarcoma of the ovary and fallopian tube. Am. J. Surg. Pathol..

[73] de Kock, L., et al. Ovarian embryonal rhabdomyosarcoma is a rare manifestation of the DICER1 syndrome. *Hum. Pathol.*10.1101/011304, 917–922 (2014). 10.1016/j.humpath.2015.02.008 25836323

[74] Apellaniz-Ruiz M, McCluggage WG, Foulkes WD (2021). DICER1-associated embryonal rhabdomyosarcoma and adenosarcoma of the gynecologic tract: Pathology, molecular genetics, and indications for molecular testing. Genes Chromosomes Cancer.

[75] Keenan RA (2019). Paratesticular sarcomas: a case series and literature review. Ther. Adv. Urol..

[76] Srigley JR (2000). The paratesticular region: histoanatomic and general considerations. Semin. Diagn. Pathol..

[77] Agarwal PK, Palmer JS (2006). Testicular and paratesticular neoplasms in prepubertal males. J. Urol..

[78] Apellaniz-Ruiz M (2020). DICER1 screening in 15 paediatric paratesticular sarcomas unveils an unusual DICER1-associated sarcoma. J. Pathol. Clin. Res..

[79] Warren M (2020). Expanding the spectrum of DICER1-associated sarcomas. Mod. Pathol..

[80] Wagh PK (2015). Cell- and developmental stage-specific DICER1 ablation in the lung epithelium models cystic pleuropulmonary blastoma. J. Pathol..

[81] Nagalakshmi VK (2011). Dicer regulates the development of nephrogenic and ureteric compartments in the mammalian kidney. Kidney Int..

[82] Pastorelli LM (2009). Genetic analyses reveal a requirement for DICER1 in the mouse urogenital tract. Mamm. Genome.

[83] Delahunt B (1993). Familial cystic nephroma and pleuropulmonary blastoma. Cancer.

[84] Joshi VV, Beckwith JB (1989). Multilocular cyst of the kidney (cystic nephroma) and cystic, partially differentiated nephroblastoma. Terminology and criteria for diagnosis. Cancer.

[85] Kajani N. R. B. & Bernstein J. Multilocular cystic nephroma. *J. Urol. Pathol*. **1**, 33–42 (1993).

[86] van den Hoek J, de Krijger R, van de Ven K, Lequin M, van den Heuvel-Eibrink MM (2009). Cystic nephroma, cystic partially differentiated nephroblastoma and cystic Wilms’ tumor in children: a spectrum with therapeutic dilemmas. Urol. Int..

[87] van Peer, S. E. et al. Clinical and molecular characteristics and outcome of cystic partially differentiated nephroblastoma and cystic nephroma: a narrative review of the literature. *Cancers* **13**, 997 (2021). 10.3390/cancers13050997 PMC7957568 33673661

[88] Li Y, Pawel BR, Hill DA, Epstein JI, Argani P (2017). Pediatric cystic nephroma is morphologically, immunohistochemically, and genetically distinct from adult cystic nephroma. Am. J. Surg. Pathol..

[89] Khan NE (2018). Structural renal abnormalities in the DICER1 syndrome: a family-based cohort study. Pediatr. Nephrol..

[90] Boman F (2006). Familial association of pleuropulmonary blastoma with cystic nephroma and other renal tumors: a report from the International Pleuropulmonary Blastoma Registry. J. Pediatr..

[91] Doros LA (2014). DICER1 mutations in childhood cystic nephroma and its relationship to DICER1-renal sarcoma. Mod. Pathol..

[92] Bahubeshi A (2010). Germline DICER1 mutations and familial cystic nephroma. J. Med. Genet..

[93] Vujanić GM, Kelsey A, Perlman EJ, Sandstedt B, Beckwith JB (2007). Anaplastic sarcoma of the kidney: a clinicopathologic study of 20 cases of a new entity with polyphenotypic features. Am. J. Surg. Pathol..

[94] Faria PA, Claudia M, Zerbini N (1996). Dedifferentiated cystic nephroma with malignant mesenchymoma as the dedifferentiated component. Pediatr. Pathol. Lab. Med..

[95] Delahunt B (1998). Cystic embryonal sarcoma of kidney: a case report. Cancer.

[96] Sola JE (2007). Primary renal botryoid rhabdomyosarcoma: diagnosis and outcome. J. Pediatr. Surg..

[97] Raney B (2008). Primary renal sarcomas in the Intergroup Rhabdomyosarcoma Study Group (IRSG) experience, 1972-2005: a report from the Children’s Oncology Group. Pediatr. Blood Cancer.

[98] Wu MK (2017). Anaplastic sarcomas of the kidney are characterized by DICER1 mutations. Mod. Pathol..

[99] Maciaszek JL, Oak N, Nichols KE (2020). Recent advances in Wilms’ tumor predisposition. Hum. Mol. Genet..

[100] MdZin R, Murch A, Charles A (2011). Pathology, genetics and cytogenetics of Wilms’ tumour. Pathology.

[101] Rivera MN, Haber DA (2005). Wilms’ tumour: connecting tumorigenesis and organ development in the kidney. Nat. Rev. Cancer.

[102] Wu MK (2013). Biallelic DICER1 mutations occur in Wilms tumours. J. Pathol..

[103] Achatz MI (2017). Cancer screening recommendations and clinical management of inherited gastrointestinal cancer syndromes in childhood. Clin. Cancer Res..

[104] Valle L (2019). Update on genetic predisposition to colorectal cancer and polyposis. Mol. Asp. Med..

[105] Apellaniz-Ruiz M (2019). Mesenchymal hamartoma of the liver and DICER1 syndrome. N. Engl. J. Med..

[106] Kapur RP, Berry JE, Tsuchiya KD, Opheim KE (2014). Activation of the chromosome 19q microRNA cluster in sporadic and androgenetic-biparental mosaicism–associated hepatic mesenchymal hamartoma. Pediatr. Dev. Pathol..

[107] Martins-Filho SN, Putra J (2020). Hepatic mesenchymal hamartoma and undifferentiated embryonal sarcoma of the liver: a pathologic review. Hepat. Oncol..

[108] Vargas SO, Perez-Atayde AR (2019). Mesenchymal hamartoma of the liver and DICER1 syndrome. N. Engl. J. Med..

[109] Putra J, Ornvold K (2015). Undifferentiated embryonal sarcoma of the liver: a concise review. Arch. Pathol. Lab. Med..

[110] See, S. C., Wadhwani, N. R., Yap, K. L. & Arva, N. C. Primary biphasic hepatic sarcoma in *DICER1* syndrome. *Pediatr. Dev. Pathol.*10.1177/10935266211008443, 10935266211008443 (2021). 10.1177/10935266211008443 33872107

[111] Larsen Haidle, J. & Howe, J. R. Juvenile polyposis syndrome. In: *GeneReviews((R))* (eds. Adam, M. P. et al.) (Seattle, WA, 1993). 20301642

[112] Jelsig AM (2014). Hamartomatous polyposis syndromes: a review. Orphanet. J. Rare Dis..

[113] Gilad O (2019). Clinical andhistologic overlap and distinction among various hamartomatous polyposis syndromes. Clin. Transl. Gastroenterol..

[114] Lallier M (1999). Pleuropulmonary blastoma: a rare pathology with an even rarer presentation. J. Pediatr. Surg..

[115] Lucia-Casadonte C (2013). An unusual case of pleuropulmonary blastoma in a child with jejunal hamartomas. Case Rep. Pediatr..

[116] Priest JR, Williams GM, Hill DA, Dehner LP, Jaffé A (2009). Pulmonary cysts in early childhood and the risk of malignancy. Pediatr. Pulmonol..

[117] Schultz KAP (2020). Pleuropulmonary blastoma-like peritoneal sarcoma: a newly described malignancy associated with biallelic DICER1 pathogenic variation. Mod. Pathol..

[118] Nakano Y (2019). Presacral malignant teratoid neoplasm in association with pathogenic DICER1 variation. Mod. Pathol..

[119] McCluggage WG (2020). Embryonal rhabdomyosarcoma of the ovary and fallopian tube: rare neoplasms associated with germline and somatic DICER1 mutations. Am. J. Surg. Pathol..

[120] Sebire NJ, Fowler D, Ramsay AD (2004). Sacrococcygeal tumors in infancy and childhood; a retrospective histopathological review of 85 cases. Fetal Pediatr. Pathol..

[121] McKenney JK, Heerema-McKenney A, Rouse RV (2007). Extragonadal germ cell tumors: a review with emphasis on pathologic features, clinical prognostic variables, and differential diagnostic considerations. Adv. Anat. Pathol..

[122] Scheckel CJ, Kosiorek HE, Butterfield R, Ho TH, Hilal T (2019). Germ cell tumors with malignant somatic transformation: a Mayo Clinic experience. Oncol. Res. Treat..

[123] Knobel M (2016). Etiopathology, clinical features, and treatment of diffuse and multinodular nontoxic goiters. J. Endocrinol. Invest..

[124] Bignell GR (1997). Familial nontoxic multinodular thyroid goiter locus maps to chromosome 14q but does not account for familial nonmedullary thyroid cancer. Am. J. Hum. Genet..

[125] Capon F (2000). Mapping a dominant form of multinodular goiter to chromosome Xp22. Am. J. Hum. Genet..

[126] Rio Frio T (2011). DICER1 mutations in familial multinodular goiter with and without ovarian Sertoli-Leydig cell tumors. JAMA.

[127] de Kock L (2016). Deep sequencing reveals spatially distributed distinct hot spot mutations in DICER1-related multinodular goiter. J. Clin. Endocrinol. Metab..

[128] Khan NE (2017). Quantification of thyroid cancer and multinodular goiter risk in the DICER1 syndrome: a family-based cohort study. J. Clin. Endocrinol. Metab..

[129] de Kock L (2014). Exploring the association between DICER1 mutations and differentiated thyroid carcinoma. J. Clin. Endocrinol. Metab..

[130] Mathews JD (2013). Cancer risk in 680,000 people exposed to computed tomography scans in childhood or adolescence: data linkage study of 11 million Australians. BMJ.

[131] Schonfeld SJ, Lee C, Berrington de González A (2011). Medical exposure to radiation and thyroid cancer. Clin. Oncol..

[132] Oue T, Inoue M, Kubota A, Kuwae Y, Kawa K (2008). Pediatric thyroid cancer arising after treatment for pleuropulmonary blastoma. Pediatr. Blood Cancer.

[133] Rome A, Gentet J-C, Coze C, André N (2008). Pediatric thyroid cancer arising as a fourth cancer in a child with pleuropulmonary blastoma. Pediatr. Blood Cancer.

[134] Shin SH (2012). Follicular thyroid carcinoma arising after hematopoietic stem cell transplantation in a child with pleuropulmonary blastoma. Thyroid.

[135] Priest JR (2010). Ciliary body medulloepithelioma: four cases associated with pleuropulmonary blastoma–a report from the International Pleuropulmonary Blastoma Registry. Br. J. Ophthalmol..

[136] Rutter MM (2016). DICER1 mutations and differentiated thyroid carcinoma: evidence of a direct association. J. Clin. Endocrinol. Metab..

[137] Guilmette J, Nose V (2018). Hereditary and familial thyroid tumours. Histopathology.

[138] Capezzone M (2020). Long-term clinical outcome in familial and sporadic papillary thyroid carcinoma. Eur. Thyroid J..

[139] Aschebrook-Kilfoy B, Grogan RH, Ward MH, Kaplan E, Devesa SS (2013). Follicular thyroid cancer incidence patterns in the United States,1980-2009. Thyroid.

[140] Song YS (2017). Changes in the clinicopathological characteristics and genetic alterations of follicular thyroid cancer. Eur. J. Endocrinol..

[141] Yoo S-K (2016). Comprehensive analysis of the transcriptional and mutational landscape of follicular and papillary thyroid cancers. PLoS Genet..

[142] Lee YA (2020). Predominant DICER1 pathogenic variants in pediatric follicular thyroid carcinomas. Thyroid.

[143] Golpanian S (2015). Pediatric papillary thyroid carcinoma: outcomes and survival predictors in 2504 surgical patients. Pediatr. Surg. Int..

[144] Wasserman JD (2018). DICER1 mutations are frequent in adolescent-onset papillary thyroid carcinoma. J. Clin. Endocrinol. Metab..

[145] Grossman RL (2016). Toward a shared vision for cancer genomic data. N. Engl. J. Med..

[146] Ibrahimpasic T (2014). Outcomes in patients with poorly differentiated thyroid carcinoma. J. Clin. Endocrinol. Metab..

[147] Asioli S (2010). Poorly differentiated carcinoma of the thyroid: validation of the Turin proposal and analysis of IMP3 expression. Mod. Pathol..

[148] Chernock RD (2020). Poorly differentiated thyroid carcinoma of childhood and adolescence: a distinct entity characterized by DICER1 mutations. Mod. Pathol..

[149] Thompson LDR, Rosai J, Heffess CS (2000). Primary thyroid teratomas. Cancer.

[150] Alexander VRC (2015). Head and neck teratomas in children—A series of 23 cases at Great Ormond Street Hospital. Int. J. Pediatr. Otorhinolaryngol..

[151] Riedlinger WFJ, Lack EE, Robson CD, Rahbar R, Nosé V (2005). Primary thyroid teratomas in children. Am. J. Surg. Pathol..

[152] Kao C-S, Bangs CD, Aldrete G, Cherry AM, Ulbright TM (2018). A clinicopathologic and molecular analysis of 34 mediastinal germ cell tumors suggesting different modes of teratoma development. Am. J. Surg. Pathol..

[153] Poulos C, Cheng L, Zhang S, Gersell DJ, Ulbright TM (2006). Analysis of ovarian teratomas for isochromosome 12p: evidence supporting a dual histogenetic pathway for teratomatous elements. Mod. Pathol..

[154] Starling, C. E., Sabra, J., Brady, B., Horton, M. & Traweek, S. T. Malignant teratoma of the thyroid: a difficult diagnosis by fine‐needle aspiration. *Diagn. Cytopathol.*10.1002/dc.24216 (2019). 10.1002/dc.24216 31120625

[155] Rooper LM (2020). Recurrent DICER1 hotspot mutations in malignant thyroid gland teratomas. Am. J. Surg. Pathol..

[156] Agaimy A (2020). Malignant teratoid tumor of the thyroid gland: an aggressive primitive multiphenotypic malignancy showing organotypical elements and frequent DICER1 alterations-is the term “thyroblastoma” more appropriate?. Virchows Arch..

[157] McDermott MB, Ponder TB, Dehner LP (1998). Nasal chondromesenchymal hamartoma: an upper respiratory tract analogue of the chest wall mesenchymal hamartoma. Am. J. Surg. Pathol..

[158] Mason KA, Navaratnam A, Theodorakopoulou E, Chokkalingam PG (2015). Nasal chondromesenchymal hamartoma (NCMH): a systematic review of the literature with a new case report. J. Otolaryngol. Head Neck Surg..

[159] Ozolek JA, Carrau R, Barnes EL, Hunt JL (2005). Nasal chondromesenchymal hamartoma in older children and adults: series and immunohistochemical analysis. Arch. Pathol. Lab. Med..

[160] Thirunavukkarasu B, Chatterjee D, Mohindra S, Dass Radotra B, Prashant SJ (2020). Nasal chondromesenchymal hamartoma. Head Neck Pathol..

[161] Priest JR, Williams GM, Mize WA, Dehner LP, McDermott MB (2010). Nasal chondromesenchymal hamartoma in children with pleuropulmonary blastoma—a report from the International Pleuropulmonary Blastoma Registry. Int. J. Pediatr. Otorhinolaryngol..

[162] Johnson C, Nagaraj U, Esguerra J, Wasdahl D, Wurzbach D (2006). Nasal chondromesenchymal hamartoma: radiographic and histopathologic analysis of a rare pediatric tumor. Pediatr. Radio.

[163] Stewart DR (2014). Nasal chondromesenchymal hamartomas arise secondary to germline and somatic mutations of DICER1 in the pleuropulmonary blastoma tumor predisposition disorder. Hum. Genet..

[164] Priest JR (2007). Cerebral metastasis and other central nervous system complications of pleuropulmonary blastoma. Pediatr. Blood Cancer.

[165] de Kock L, Priest JR, Foulkes WD, Alexandrescu S (2019). An update on the central nervous system manifestations of DICER1 syndrome. Acta Neuropathol..

[166] Kaneko H (2011). DICER1 deficit induces Alu RNA toxicity in age-related macular degeneration. Nature.

[167] Huryn LA (2019). DICER1 syndrome: characterization of the ocular phenotype in a family-based cohort study. Ophthalmology.

[168] Kaliki S (2013). Ciliary body medulloepithelioma. Ophthalmology.

[169] Shields JA, Eagle RC, Ferguson K, Shields CL (2015). Tumors of the nonpigmented epithelium of the ciliary body. Retina.

[170] Broughton WL, Zimmerman LE (1978). A clinicopathologic study of 56 cases of intraocular medulloepitheliomas. Am. J. Ophthalmol..

[171] Shields JA, Eagle RC, Shields CL, De Potter P (1996). Congenital neoplasms of the nonpigmented ciliary epithelium (medulloepithelioma). Ophthalmology.

[172] Zimmerman LE (1970). The remarkable polymorphism of tumours of the ciliary epithelium. Trans. Aust. Coll. Ophthalmol..

[173] Kramer, G. D., Arepalli, S., Shields, C. L. & Shields, J. A. Ciliary body medulloepithelioma association with pleuropulmonary blastoma in a familial tumor predisposition syndrome. *J. Pediatr. Ophthalmol. Strabismus.*10.3928/01913913-20140709-03 (2014). 10.3928/01913913-20140709-03 25032694

[174] Durieux E, Descotes F, Nguyen A-M, Grange JD, Devouassoux-Shisheboran M (2015). Somatic DICER1 gene mutation in sporadic intraocular medulloepithelioma without pleuropulmonary blastoma syndrome. Hum. Pathol..

[175] Scheithauer BW (2011). Pituitary blastoma: a unique embryonal tumor. Pituitary.

[176] Scheithauer BW (2008). Pituitary blastoma. Acta Neuropathol..

[177] de Kock L (2014). Pituitary blastoma: a pathognomonic feature of germ-line DICER1 mutations. Acta Neuropathol..

[178] Kivelä T (1999). Trilateral retinoblastoma: a meta-analysis of hereditary retinoblastoma associated with primary ectopic intracranial retinoblastoma. J. Clin. Oncol..

[179] de Kock L (2014). Germline and somatic DICER1 mutations in pineoblastoma. Acta Neuropathol..

[180] Sabbaghian N (2012). Germline DICER1 mutation and associated loss of heterozygosity in a pineoblastoma. J. Med. Genet..

[181] Das A (2019). Germline DICER1‐mutant intracranial sarcoma with dual chondroid and spindle cell morphology and pulmonary metastases treated with multimodal therapy. Pediatr. Blood Cancer.

[182] Kamihara J (2020). DICER1-associated central nervous system sarcoma in children: comprehensive clinicopathologic and genetic analysis of a newly described rare tumor. Mod. Pathol..

[183] Koelsche C (2018). Primary intracranial spindle cell sarcoma with rhabdomyosarcoma-like features share a highly distinct methylation profile and DICER1 mutations. Acta Neuropathol..

[184] Lee JC (2019). Primary intracranial sarcomas with DICER1 mutation often contain prominent eosinophilic cytoplasmic globules and can occur in the setting of neurofibromatosis type 1. Acta Neuropathol..

[185] Sakaguchi M (2019). Two cases of primary supratentorial intracranial rhabdomyosarcoma with DICER1 mutation which may belong to a “spindle cell sarcoma with rhabdomyosarcoma-like feature, DICER1 mutant”. Brain Tumor Pathol..

[186] Uro-Coste E (2018). ETMR-like infantile cerebellar embryonal tumors in the extended morphologic spectrum of DICER1-related tumors. Acta Neuropathol..

[187] Khan NE (2017). Macrocephaly associated with the DICER1 syndrome. Genet. Med..

[188] Cao H (2010). MicroRNAs play a critical role in tooth development. J. Dent. Res..

[189] Michon F, Tummers M, Kyyrönen M, Frilander MJ, Thesleff I (2010). Tooth morphogenesis and ameloblast differentiation are regulated by micro-RNAs. Dev. Biol..

[190] Oommen S (2012). Distinct roles of MicroRNAs in epithelium and mesenchyme during tooth development. Dev. Dyn..

[191] Choi S (2019). Dental abnormalities in individuals with pathogenic germline variation in DICER1. Am. J. Med. Genet. A.

[192] Klein S (2014). Expanding the phenotype of mutations in DICER1: mosaic missense mutations in the RNase IIIb domain of DICER1 cause GLOW syndrome. J. Med. Genet..

[193] Knudson AG (1971). Mutation and cancer: statistical study of retinoblastoma. Proc. Natl Acad. Sci. USA.

[194] Mendoza PR, Grossniklaus HE (2015). The biology of retinoblastoma. Prog. Mol. Biol. Transl. Sci..

[195] McEvoy JD, Dyer MA (2015). Genetic and epigenetic discoveries in human retinoblastoma. Crit. Rev. Oncog..

[196] Byrjalsen A (2020). Nationwide germline whole genome sequencing of 198 consecutive pediatric cancer patients reveals a high incidence of cancer prone syndromes. PLoS Genet..

[197] Capasso M (2020). Genetic predisposition to solid pediatric cancers. Front. Oncol..

[198] Valdez JM, Nichols KE, Kesserwan C (2017). Li-Fraumeni syndrome: a paradigm for the understanding of hereditary cancer predisposition. Br. J. Haematol..

[199] Theotoki, E. I. et al. Dicing the disease with DICER: the implications of DICER ribonuclease in human pathologies. *Int. J. Mol. Sci.* **21**, 1–24 (2020). 10.3390/ijms21197223 PMC7583940 33007856

[200] Pong SK, Gullerova M (2018). Noncanonical functions of microRNA pathway enzymes - Drosha, DGCR8, Dicer and Ago proteins. FEBS Lett..

